# Distinct Tissue‐Dependent Composition and Gene Expression of Human Fetal Innate Lymphoid Cells

**DOI:** 10.1002/eji.202451150

**Published:** 2024-12-15

**Authors:** Inga E. Rødahl, Martin A. Ivarsson, Liyen Loh, Jeff E. Mold, Magnus Westgren, Danielle Friberg, Jenny Mjösberg, Niklas K. Björkström, Nicole Marquardt, Douglas F. Nixon, Jakob Michaëlsson

**Affiliations:** ^1^ Center for Infectious Medicine Department of Medicine Huddinge Karolinska Institutet Karolinska University Hospital Stockholm Sweden; ^2^ Division of Experimental Medicine Department of Medicine University of California San Francisco San Francisco USA; ^3^ Department of Cell and Molecular Biology Karolinska Institutet Stockholm Sweden; ^4^ Center for Fetal Medicine Department of Clinical Science Intervention and Technology Karolinska Institutet Stockholm Sweden; ^5^ Department of Surgical Sciences Uppsala University Uppsala Sweden; ^6^ Clinical Lung‐ and Allergy Research Unit Medical Unit for Lung and Allergy Diseases Karolinska University Hospital Huddinge Stockholm Sweden; ^7^ Center for Hematology and Regenerative Medicine Department of Medicine Huddinge Karolinska Institutet Stockholm Sweden

## Abstract

The human fetal immune system starts to develop in the first trimester and likely plays a crucial role in fetal development and maternal‐fetal tolerance. Innate lymphoid cells (ILCs) are the earliest lymphoid cells to arise in the human fetus. ILCs consist of natural killer (NK) cells, ILC1s, ILC2s, and ILC3s that all share a common lymphoid origin. Here, we studied fetal ILC subsets, mainly NK cells and ILC3s and their potential progenitors, across human fetal tissues. Our results show that fetal ILC subsets have distinct distribution, developmental kinetics, and gene expression profiles across human fetal tissues. Furthermore, we identify CD34^+^RORγt^+^Eomes^−^ and CD34^+^RORγt^+^Eomes^+^ cells in the fetal intestine, indicating that tissue‐specific ILC progenitors exist already during fetal development.

## Graphical abstract

CD16^+^ and CD16^−^ Natural Killer cells and type 3 innate lymphoid cells are found in early human development with distinct distribution and phenotype across fetal tissues. The presence of CD34^+^RORyt^+^Eomes^±^ cells selectively in the fetal intestine indicates the presence of tissue‐specific progenitors.

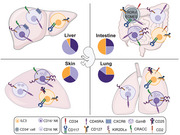

**Abbreviations**
BM: bone marrow
CILP: common innate lymphoid progenitor
ILC: innate lymphoid cell
ILCP: innate lymphoid cell progenitor
LTi: lymphoid tissue inducer
NK: natural killer
PCW: postconception week

## 1 Introduction

Natural killer (NK) cells and other innate lymphoid cells (ILCs) are rapidly responding immune cells found in lymphoid and nonlymphoid tissues [1]. ILCs are divided into three major groups depending on their function and expression of transcription factors. Group 1 ILCs consist of both cytotoxic‐ and IFN‐γ‐producing Eomes^+^Tbet^+/−^ NK cells [2] and IFN‐γ‐producing Eomes^−^Tbet^+^ ILC1 [3]. Group 2 ILCs consists of IL‐5 and IL‐13 producing GATA‐3^+^ ILC2s [4, 5]. Finally, group 3 ILCs consists of IL‐17 and IL‐22 producing RORγt^+^ ILC3s [6, 7, 8, 9]. Group 3 ILCs also include lymphotoxin and TNF‐producing lymphoid tissue inducer (LTi) cells [10, 11], which are important for lymphoid tissue formation in mice. However, the distinction between LTi cells and other ILC3s is less clear in humans [1]. ILCs are also commonly identified by the expression of combinations of cell surface proteins. Most studies define NK cells as Lin^−^CD56^+^CD127^−^ cells and they are commonly divided into CD56^bright^ (CD16^−^) and CD56^dim^ (CD16^+^) NK cells. Non‐NK cell ILCs are defined as Lin^−^CD127^+^ cells, among which ILC1s are defined as Lin^−^CD127^+^CRTH2^−^CD117^−^NKp44^+/−^ cells, ILC2 as Lin^−^CD127^+^CRTH2^+^ cells, and ILC3s as Lin^−^CD127^+^ CRTH2^−^CD117^+^NKp44^+/−^ cells. Variations in the definitions of ILC subsets between studies, including the use of different lineage markers, have complicated comparisons of results across studies. For example, CD7 [12, 13] and CD11b [14] expressions have been used either as an inclusion or exclusion criterion in some studies, whereas in other studies neither is used.

NK cells, ILC2s, and ILC3s can be detected in fetal tissues already during the first trimester [6, 14, 15, 16], whereas ILC1s appear to develop later, possibly after birth [3]. NK cells have been detected in fetal liver and skin as early as a postconception week (PCW) 6 [15, 16, 17], and functional CD16^−^ and CD16^+^ NK cells capable of responding to target cells and cytokines are present in second‐trimester fetal tissues [18, 19]. In addition to NK cells, ILC3s are abundant in fetal tissues [12, 14, 16, 17], with considerable heterogeneity in terms of both protein and RNA expression [12, 14, 17]. For example, ILC3s with tissue‐resident characteristics seem to develop in second‐trimester intestines and lungs [20], and CD304^+^ ILC3s are abundant in fetal and adult lymphoid tissues. CD304^+^ ILC3s are therefore of particular interest given their suggested role as LTi cells [21]. However, most studies of human fetal ILCs have focused on a single tissue or a single type of ILC, and there is limited data on how the different ILCs develop over time across different fetal tissues.

Generally, NK cells predominate among ILCs in most adult tissues studied, including the liver, lung, uterus, spleen, and blood [22, 23, 24], although ILC3s are enriched in the intraepithelial compartment of the ileum [25]. In addition, the different ILC subsets have distinct gene expression profiles in different adult tissues [25, 26], potentially reflecting subset‐specific migratory patterns and/or tissue‐specific imprinting. To what extent defined human fetal ILC subsets have distinct gene expression profiles has not been analyzed in detail, although differences in the composition of transcriptionally defined ILC subsets between tissues have been reported [14].

Several studies have explored the stages at which different groups of ILCs diverge from each other [13, 14, 27, 28, 29, 30]. A fraction of the common innate lymphoid progenitor (CILP) cells, defined as CD34^+^CD45RA^+^CD117^+^IL1R1^+^RORγt^+^, in tonsils, can generate all groups of ILCs, including NK cells [29]. Similarly, a fraction of CD34^−^CD117^+^CD127^+^RORγt^−^ cells (ILCPs) in cord blood, and adult blood and lung can differentiate into all groups of ILCs [13], indicating that the divergence of ILC subsets may occur late in maturation. Downstream of CILPs and ILCPs, CD34^−^CD117^+^CD56^+^ cells in the tonsil can give rise to both NK cells and ILC3s, but not ILC2s [31]. However, CD34^+^ NK cell‐restricted progenitors in cord blood, defined as CD34^+^CD45RA^+^CD7^+^CD10^+^CD127^−^ cells, have also been identified, suggesting that at least some NK cells might diverge from the common ILC development at an earlier stage [28]. There is considerable heterogeneity within each of the described progenitor populations, leaving room for more refined studies of ILC development. In addition, it remains possible that ILCs develop differently depending on tissue location in vivo, and that even phenotypically similar ILCs may develop from distinct, possibly tissue‐restricted, progenitors.

Although it is known that ILCs develop early in the human fetus, there is a lack of studies examining defined ILC subsets in parallel throughout gestation and across multiple tissues using combined analysis of key transcription factors and cell surface markers. Moreover, little is known regarding gene and protein expression differences among defined ILC subsets across fetal tissues. To address these questions, we analyzed ILC subsets and potential progenitor subsets from human fetal tissues using flow cytometry and RNA sequencing. We demonstrate distinct subset distributions and gene expression profiles of human ILCs across fetal tissues. Collectively, our findings enhance our understanding of ILC ontogeny, composition, and dynamics in various fetal tissues.

## 2 Results

### 2.1 Fetal ILCs Have Distinct Subset Composition and Developmental Kinetics Dependent on Tissue Location

**FIGURE 1:**
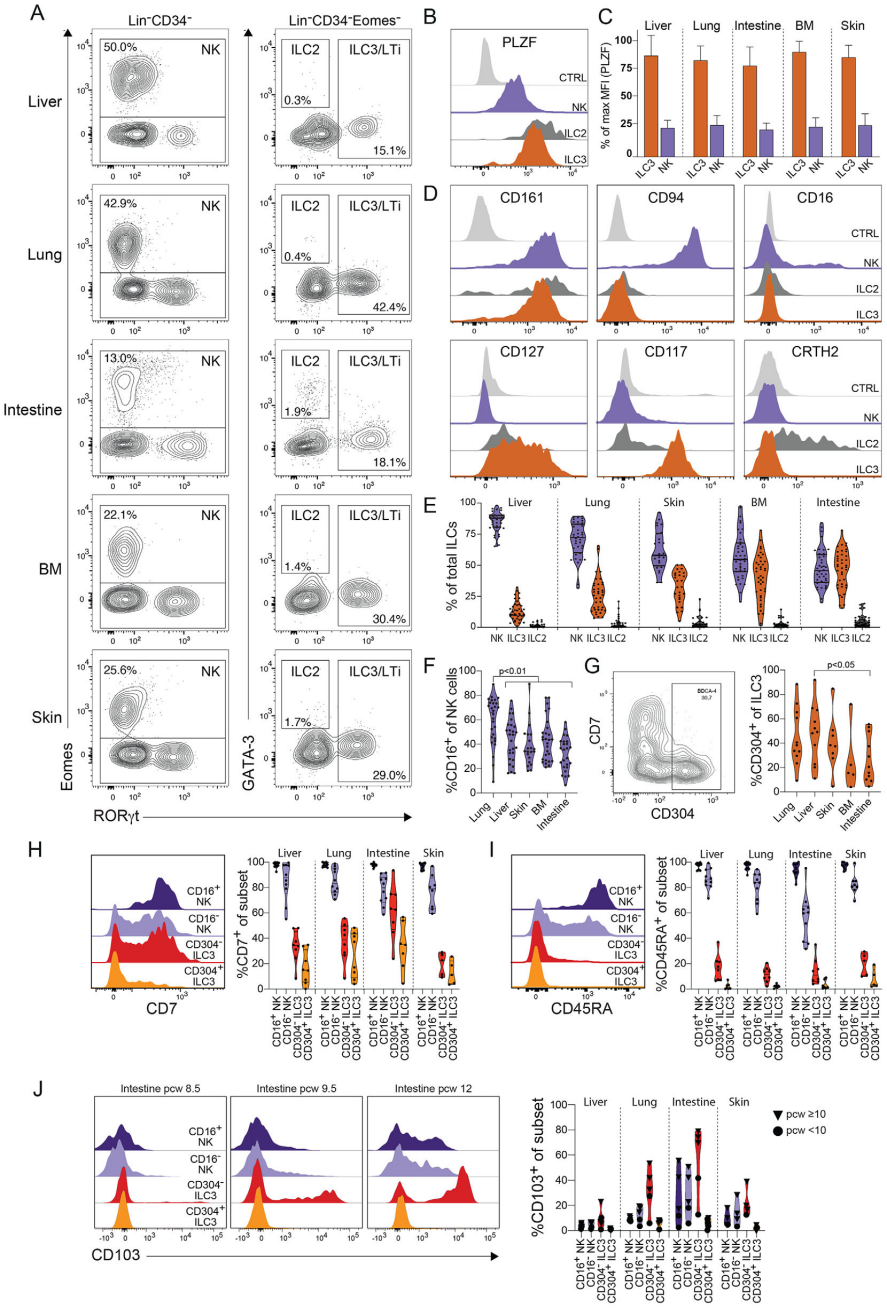
Characterization of fetal ILC subsets across tissues using flow cytometry. (A) Representative staining and gating of human NK cells (Lin^−^CD34^−^Eomes^+^), ILC2 (Lin^−^CD34^−^RORγt^−^GATA3^+^), and ILC3 (Lin^−^CD34^−^Eomes^−^RORγt^+^) in fetal liver, lung, intestine, bone marrow (BM), and skin at PCW 9.5. (B) Representative histogram showing expression of PLZF in intestinal NK cells, ILC2s, and ILC3s at PCW 9.5. Control: Lin^−^CD34^−^Eomes^−^RORγt^−^GATA3^−^CD161^− ^cells. ILC subsets are defined as in (A). (C) Relative fluorescence intensity of PLZF in ILC3s and NK cells across tissues. Bars represent mean with SD. *n* = 14 (BM), *n* = 11–13 (intestine, liver, and lung), *n* = 7–8 (skin) at PCW 8–11. (D) Representative flow cytometry histogram showing expression of CD161, CD94, CD16, CD127, CD117, and CRTH2 on NK cells, ILC2s, and ILC3s in the fetal intestine at PCW 9.5. (E) Violin plots showing the frequency of ILC subsets of total ILCs across fetal liver, lung, skin, BM, and intestine. Bars indicate mean, *n* = 38–40, *n* = 27 (skin only), PCW 6–20. (F) Violin plots showing the frequency of CD16^+^ NK cells among total NK cells across tissues. Statistical analysis using one‐way related measures ANOVA, *n* = 39–40 (liver and lung), *n* = 35–36 (intestine, BM), *n* = 26 (skin), PCW 7–20. (G) Representative staining of CD304 and CD7 on ILC3s (middle, defined as in A) at PCW 10, and frequency of CD304^+^ ILC3 of total ILC3s across tissues (right) (PCW 7.5‐12). Statistical analysis using one‐way related measures ANOVA, paired tissues *n* = 11 (liver, lung, and gut), *n* = 8 (skin), *n* = 5 (BM). (H–I) Representative histograms and frequencies of (G) CD7^+^ cells and (H) CD45RA^+^ cells, among CD16^+^ and CD16^−^ NK cells and CD304^−^ and CD304^+^ ILC3s (*n* = 5–10, PCW 8–10). (J) Representative histograms and frequencies of CD103^+^ cells across tissues and gestational age among CD16^+^ and CD16^−^ NK cells and CD304^−^ and CD304^+^ ILC3s (*n* = 5).

We first analyzed human fetal ILCs in the liver, lung, intestine, bone marrow (BM), and skin, primarily during the first trimester of gestation. Using flow cytometry, we analyzed the expression of transcription factors important for ILC development and function [1], including Eomes, Tbet, RORγt, GATA‐3, and PLZF, together with the expression of cell surface receptors. We identified NK cells as Lin^−^(CD3^−^CD14^−^CD19^−^)CD34^−^Eomes^+^ cells, ILC3s as Lin^−^CD34^−^Eomes^−^RORγt^+^ cells, and ILC2s as Lin^−^CD34^−^Eomes^−^RORγt^−^GATA‐3^+^ cells (Figure 1A; Figure S1A). Consistent with previous studies [3], virtually no bona fide ILC1 cells defined as Lin^−^CD34^−^Tbet^+^Eomes^−^CD127^+^ could be identified, while Tbet expression was readily detected in Eomes^+^ NK cells (Figure S1B). In contrast to mice [32], and consistent with studies of adult ILCs [31, 33], all fetal ILC subsets expressed the transcription factor PLZF in all tissues analyzed, albeit at higher levels in ILC2s and ILC3s compared with NK cells (Figure 1B,C).

The ILC subsets identified by analyses of transcription factor expression had a cell surface phenotype consistent with existing definitions [1]. Virtually all ILCs identified in our analysis expressed CD161. All NK cells expressed CD94, and a fraction expressed CD16, whereas these markers were not expressed by other ILCs. Both ILC2s and ILC3s expressed IL‐7 receptor (CD127), ILC3s expressed CD117, and the majority of ILC2s expressed the prostaglandin receptor CRTH2 (Figure 1D). However, in contrast to adult NK cells [34], only a subset of fetal NK cells expressed the activating receptor NKp80 (Figure S1C). Similarly, the average frequency of NKp44^+^ cells among NK cells in all tissues and among ILC3s in the liver, skin, and BM was below 10%. However, a significantly larger fraction of ILC3s in the intestine and lung expressed NKp44 compared with the liver (Figure S1D).

NK cells and ILC3s were the dominant ILC subpopulations across all tissues, whereas ILC2s constituted less than 10% of the total ILCs (Figure 1E). NK cells made up the majority of ILCs in the liver, lung, skin, and BM, whereas NK cells and ILC3s were equally frequent in the intestine (Figure 1E). The frequency of NK cells among total ILCs increased significantly over gestation in the liver, lung, and BM at the expense of a decrease in ILC3s, whereas no significant changes could be detected over time in the intestine and skin (Figure S1E). Confirming previous studies [15, 16, 35], ILCs developed several weeks before T cells could be detected in peripheral tissues (Figure S1F).

Given the variation in NK cell and ILC3 frequencies between fetal tissues, we next determined whether the composition of each ILC subset also varied. NK cells are commonly divided into CD16^−^ and CD16^+^ NK cells, with distinct functions and tissue distribution [36]. Similarly, CD304^+^ and CD304^−^ ILC3s have distinct tissue distribution and gene expression profiles [21]. The frequency of CD16^+^ NK cells was highest in the lung (Figure 1F), and the frequency of CD304^+^ ILC3s was highest in the liver (Figure 1G), while the frequencies of both subsets were lowest in the intestine (Figure 1F,G). We did not detect any significant time‐dependent changes in the frequency of CD16^+^ cells among NK cells or CD304^+^ cells among ILC3s during the first trimester (Figure S1G,H).

To further dissect the heterogeneity within each of the subsets across tissues, we analyzed the expression of markers associated with ILC differentiation (CD7 and CD45RA) [33, 37], and tissue residency (CD103) [38]. Similar to adult NK cells, virtually all fetal CD16^+^ NK cells and the majority of fetal CD16^−^ NK cells expressed both CD7 and CD45RA in all tissues (Figure 1H,I). In contrast, very few CD304^+^ and CD304^−^ ILC3s expressed CD45RA, and only a minority expressed CD7, except for higher frequencies of CD7^+^ CD304^−^ ILC3s in the intestine (Figure 1H,I). However, we did note that the expression of CD7 on ILC3s increased with gestation (Figure S1I). The expression of CD103 varied substantially between ILC subsets, tissues, and with gestational age (Figure 1J). Up to 80% of CD304^−^ ILC3s in the intestine and 30% of CD304^−^ ILC3s in the lung expressed CD103 after PCW 10. In contrast, less than 10% of CD304^+^ ILC3s expressed CD103 in all tissues (Figure 1J). In addition, up to 50% of both CD16^+^ and CD16^−^ NK cells in the intestine expressed CD103 at later gestational ages (Figure 1J).

A large proportion of the ILCs expressed Ki67, indicative of ongoing proliferation, with the highest frequency of Ki67^+^ cells in the fetal intestine (Figure S1J). Notably, CD16^−^ NK cells and CD304^−^ ILC3s expressed Ki67 more frequently compared with CD16^+^ NK cells and CD304^+^ ILC3s (Figure S1J), in line with the notion that the latter represent more differentiated subsets of NK cells and ILC3s.

Taken together, NK cells and ILC3s make up the vast majority of ILCs during the first and second trimesters but vary in composition between tissues and over gestational age.

### 2.2 Fetal ILC Subsets Have Distinct Tissue‐Specific Transcriptional Profiles

**FIGURE 2:**
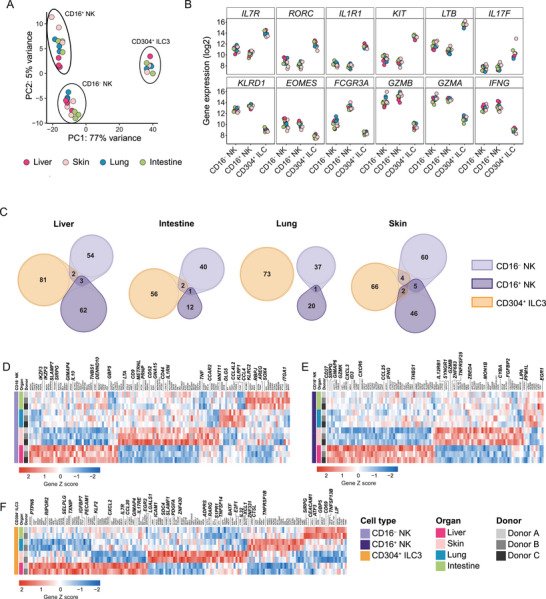
Fetal ILC subsets have tissue‐specific transcriptomic profiles. Bulk RNA analysis of CD16^−^ and CD16^+^ NK cells, and CD304^+^ ILCs from matched fetal liver, skin, intestine, and lung (PCW 9.5). (A) PCA plot showing PC1 and PC2 depicting variance of the data (B) Normalized and variance stabilized gene expression (log2) of canonical markers. (C) Euler diagram depicting the overlap of differentially upregulated genes from each subset in the same tissue. (D–F) Heatmaps of unique differentially expressed genes from pairwise comparison of fetal tissues. Genes are grouped by tissue and by expression level, visualized by z‐score. Only genes uniquely differentially upregulated by one tissue are included. (D) CD16^−^ NK cells (*n* = 3), (E) CD16^+^ NK (*n* = 3) cells, and (F) CD304^+^ ILCs (*n* = 2). Differentially expressed genes from pairwise comparisons with *p*_adj_ < 0.01 and log2 fold change > 1 are shown.

To deepen our analysis of fetal ILCs, we examined the transcriptional landscape of sorted CD16^+^ and CD16^−^ NK cells, as well as CD304^+^ ILC3s, from matched human fetal liver, intestine, skin, and lung (PCW 9.5) (see Figure S2A for gating strategy). Principal component analysis (PCA) revealed a distinct separation of the three subsets, independent of tissue (Figure 2A). The first principal component (PC1) accounted for 77% of the variance and clearly separated CD304^+^ ILC3s from the NK cell subsets, whereas PC2 accounted for 5% of the variance, separating CD16^+^ from CD16^−^ NK cells (Figure 2A).

We confirmed the consistent identity of the sorted subsets across all tissues based on the expression of canonical genes. Specifically, CD304^+^ ILC3s in all tissues expressed high levels of *IL7R*, *RORC*, *IL1R1*, *KIT*, *LTB*, *LTA*, *IL17A*, and *IL17F*, and both CD16^−^ and CD16^+^ NK cell subsets expressed high levels of *KLRD1*, *EOMES*, *GZMB*, *GZMA*, *IFNG*, and *CCL3‐5* (Figure 2B; Figure S2B–D). CD16⁺ NK cells were distinguished from CD16^−^ NK cells by high expression of *FCGR3A*, *FGFBP2*, and *ZEB2*, whereas CD16⁻ NK cells expressed slightly higher levels of *IL7R* and *KIT* (Figure 2B; Figure S2B–D). The top ten differentially expressed genes from a pairwise comparison of the three subsets further substantiated their distinct identities across all tissues (Figure S2B). In addition, all ILC subsets expressed the transcription factors *IKZF1*, *IKZF2*, *IKZF3*, *ZBTB16*, *RUNX2*, *RUNX3*, and *BATF*, which are known to regulate ILC development and function, albeit at variable levels depending on ILC subset (Figure S2C). Similarly, both NK cells and CD304^+^ ILC3s expressed genes encoding cytokines (*TNF* and *CSF2*) and chemokines (*CCL3‐5*, *CXCL2*; Figure S2D).

Having confirmed consistent differences between ILC subsets across tissues, we next compared the four different tissues for each ILC subset separately to identify tissue‐specific gene expression patterns. Pairwise differential gene expression analysis between tissues revealed 218 differentially expressed genes for CD304^+^ ILC3s, and 164 and 128 differentially expressed genes for CD16^−^ and CD16^+^ NK cells, respectively (*p*_adj_ < 0.01, log2foldchange > 1) (Figure S3A,B; Supporting Information Data 1; Table S1). To determine whether the subsets shared overlapping transcriptional tissue profiles, we compared upregulated genes from each subset in each tissue (Figure 2C; Figure S3A,B). CD16^+^ and CD16^−^ NK cells had overlapping upregulated genes in the liver (*SIRPG*, *H2BC8*, *THBS1*), skin (*KIR2DL1*, *IL12RB1*, *SYNGR1*, *METRNL*, *RASSF4*), lung (*KIR2DL1*), and intestine (*KIR2DL1*). Additionally, CD16^−^ NK cells and CD304^+^ ILC3s overlapped in their expression profiles in multiple tissues, sharing upregulated genes in the liver (*WDR3*, *GIMAP4*), in the skin (*LGALS1*, *LMNA*, *ELOA*, *ADPRS*), and the intestine (*DHRS3*, *SUSD3*). In contrast, CD16^+^ NK cells and CD304^+^ ILC3s only shared the upregulation of genes in the skin (*SLC43A1*, *TMBIM1*). Notably, there was no tissue where all three subsets had overlapping upregulation of genes (Figure 2C). This observation is in line with the PCA results, suggesting that the tissue environment does not strongly drive uniform changes in the transcriptional profile across all ILC subsets.

**FIGURE 3:**
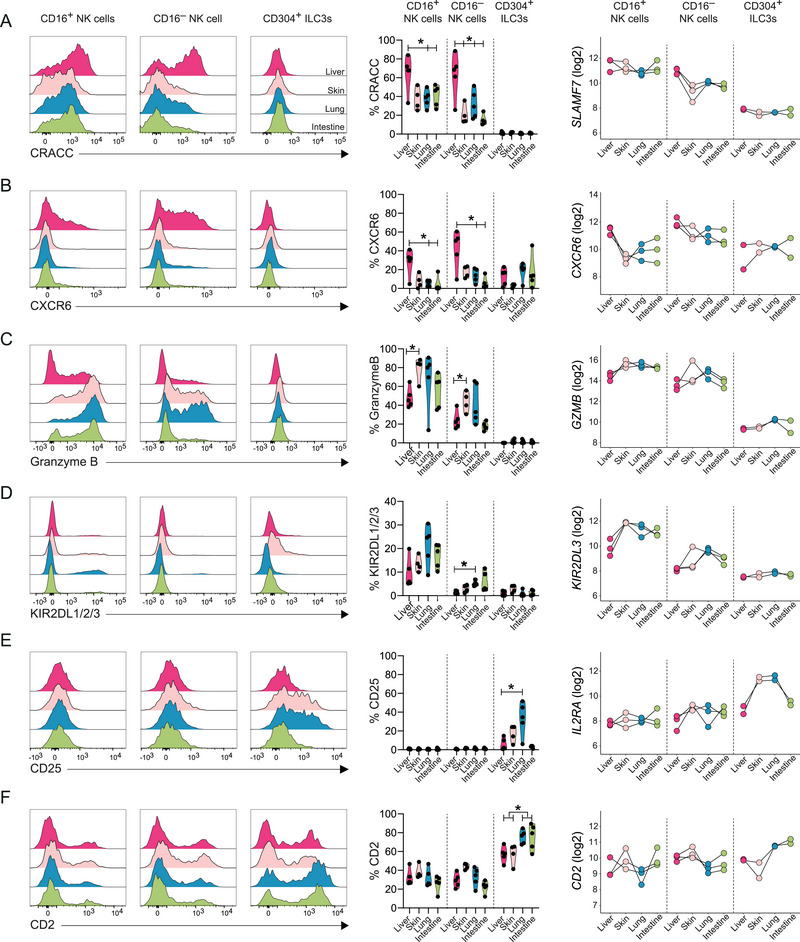
Protein expression confirms transcriptional patterns across fetal tissues. (A–F) Representative histogram plots (PCW 9.5), frequencies (PCW 8.5–12, *n* = 4–5), and RNA expression (PCW 9.5, *n* = 2–3) in fetal liver, skin, lung, and intestine by CD16^+^ and CD16^−^ NK cells and CD304^+^ ILC3s. (A) CRACC and *SLAMF7*, (B) CXCR6 and *CXCR6*, (C) Granzyme B and *GZMB*, (D) KIR2DL1/2/3 and *KIR2DL3*, (E) CD25 and *IL2RA*, and (F) CD2 and *CD2*. Statistical analysis of protein expression by mixed‐effects analysis with Geisser‐greenhouse correction and Tukey's multiple comparison test. *p_adj_ < 0.05, **p_adj_ < 0.01, ***p_adj_ < 0.001.

We next identified genes uniquely upregulated in one tissue for each separate ILC subset (Figure 2D–F). Among these genes, fetal liver CD16^−^ NK cells upregulated genes encoding cell surface receptors regulating function (*SIRPG*, *SLAMF7*), immunomodulatory secreted proteins (*IL10*, *THBS1*), and transcription factors regulating NK cell reactivity and development (*IKZF2, IKZF3*) (Figure 2D). Fetal liver CD16^+^ NK cells upregulated genes encoding a liver‐homing receptor *(CXCR6)*, effector functions (*IFNG, CSF1, GZMK*), chemokines (*CXCL3*, *CCL25*), and co‐activating receptors (*CD27*, *SIRPG*) (Figure 2E). We confirmed the high expression of the activating receptor *SLAMF7* (CRACC) by CD16^−^ NK cells in the fetal liver at the protein level compared with all other tissues (Figure 3A). In addition, CD16^+^ NK cells in the fetal liver also expressed higher levels of CRACC compared with other tissues, even though *SLAMF7* expression was consistent across tissues (Figure 3A). Similarly, we confirmed higher protein expression of CXCR6 in both CD16^+^ and CD16^−^ NK cells in the fetal liver compared with other tissues (Figure 3B), in line with previous findings [19]. Notably, CD304^+^ ILC3s did not express higher levels of CXCR6 in fetal liver (Figure 3B).

CD16^−^ NK cells in fetal skin expressed higher levels of receptors regulating adhesion and function (*CD44*, *CD52*, *CD9*), and secreted proteins (*LTA*, *METRNL*, *TNF*, *IL1RN*) (Figure 2D), while CD16^+^ NK cells expressed higher levels of cytokine receptors (*IL12RB*, *TNFRSF25*), the cytotoxicity‐related marker *FGFBP2*, and the transcription factor *ZNF683* (Figure 2E). CD16^−^ NK cells exhibited a greater number of uniquely upregulated genes in the lung and intestine compared with CD16^+^ NK cells (Figure 2D,E). For example, CD16^−^ NK cells in the fetal intestine upregulated genes associated with tissue residency (*AREG*, *RBPJ*, *SOX4*, *ITGA1*), and the activating receptor *KLRC2*, whereas lung CD16^−^ NK cells upregulated the activating receptor *KLRF1* and chemokines (*CCL4L2*, and *CCL4*) (Figure 2D). Notably, both NK cell subsets significantly upregulated multiple KIR genes in the skin, lung, and intestine in comparison to the liver (Figure S3B). Specifically, CD16^+^ NK cells upregulated *KIR2DL3* and *KIR2DS4* in the lung and skin, and *KIR3DL1* in the skin, lung, and intestine compared with the liver. In addition, CD16^−^ NK cells upregulated *KIR3DL2* in the skin and lung compared with the liver (Figure S3B). Protein expression of KIR2DL1/2/3 showed a similar trend, with higher frequencies of KIR^+^ cells in skin, lung, and intestine in both NK cell subsets, although the expression was notably higher by CD16^+^ NK cells (Figure 3D). In addition, CD16^+^ NK cells in the skin upregulated *GZMB* compared with fetal liver (Figure 2E), which was also confirmed at the protein level (Figure 3C). Similarly, there was a trend of higher RNA and protein expression of granzyme B by CD16^+^ NK cells in the fetal lung and intestine compared with the liver (Figure 3C). This extended to CD16^−^ NK cells in fetal skin, although granzyme B expression in CD16^−^ NK cells was overall lower than in CD16^+^ NK cells (Figure 3C).

CD304^+^ ILC3s displayed a higher number of differentially expressed genes unique for each tissue compared with NK cells (Figure 2F). For example, liver CD304^+^ ILC3s expressed higher levels of cytokine receptors (*IL7R, IL17RE*), chemokines (*CCL20*, *CXCL2)*, genes associated with tissue‐egress (*KLF2*, *RIPOR2*), inhibitory signaling (*PTPN6*, *PECAM1*), transcriptional regulation (*CTCF*, *ZNF488*), regulation of apoptosis (*GIMAP4*), and *IGFBP7*. In the skin, CD304^+^ ILC3s expressed higher levels of several genes encoding transcription factors relevant for ILC3 biology (*RARG*, *ZNF430*, *BATF*, *E2F1*, *EGR2*), signaling (*SLAMF1)*, and interactions with surrounding cells (*TNFSF14*, *PDGFA*, *LGALS1*, *ICAM1*). In the lung, CD304^+^ ILC3s upregulated the TNF receptor *TNFRSF1B*, the chemokine *XCL1*, and the transcription factor *ZNF331* [26]. Finally in the intestine CD304^+^ ILC3s upregulated membrane receptors (*CD38*, *SIRPG*, *CEACAM1)*, and cytokines (*TNFSF13B* and *LIF*) (Figure 3F). Although CD304^+^ ILC3s infrequently expressed the tissue‐residency marker CD103 at the protein level (Figure 1I), they upregulated genes associated with tissue‐residency in lung and intestine (*ALOX5AP*, *RGS1*, and *RGS2*) (Figure S3A). In addition, lung and intestine CD304^+^ ILC3s expressed higher levels of the activating receptors *CD2* and *NCR2* (NKp44) (Figure S3A; Figure 3E,F), in line with the reported co‐expression in adult ILC3s [39], and high expression of NKp44 in the adult intestine compared with liver [40]. Both skin and lung CD304^+^ ILC3s expressed higher levels of *IL2RA* (CD25) compared with liver and intestine, which has also been observed by adult ILC3s in skin and lung [41]. The differences in expression of *CD2* and *IL2RA* in CD304^+^ ILC3s between tissues were also confirmed at the protein level (Figure 3E,F).

In summary, each fetal ILC subset had a subset‐specific transcriptional profile independent of tissue. Simultaneously, within each subset, we also found unique tissue‐specific transcriptional profiles, including genes involved in the regulation of ILC function, migration, and development. The overall consistency that these genes exhibit between distinct fetal specimens highlights the utility of these signatures for future investigations into the biology of each distinct subset.

### 2.3 Subsets of CD34^+^ Cells in Human Fetal Intestine Express RORγt and Eomes

To explore whether the tissue‐specific gene expression patterns could be due to the development of ILCs from tissue‐restricted ILC progenitors, we assessed whether CD45^+^CD34^+^ cells in fetal tissues expressed ILC‐associated proteins. CD34^+^RORγt^+^ progenitors have previously been described as ILC‐restricted in adult tonsils [29, 30], but it remains unknown if cells with a similar phenotype exist in fetal tissues.

**FIGURE 4:**
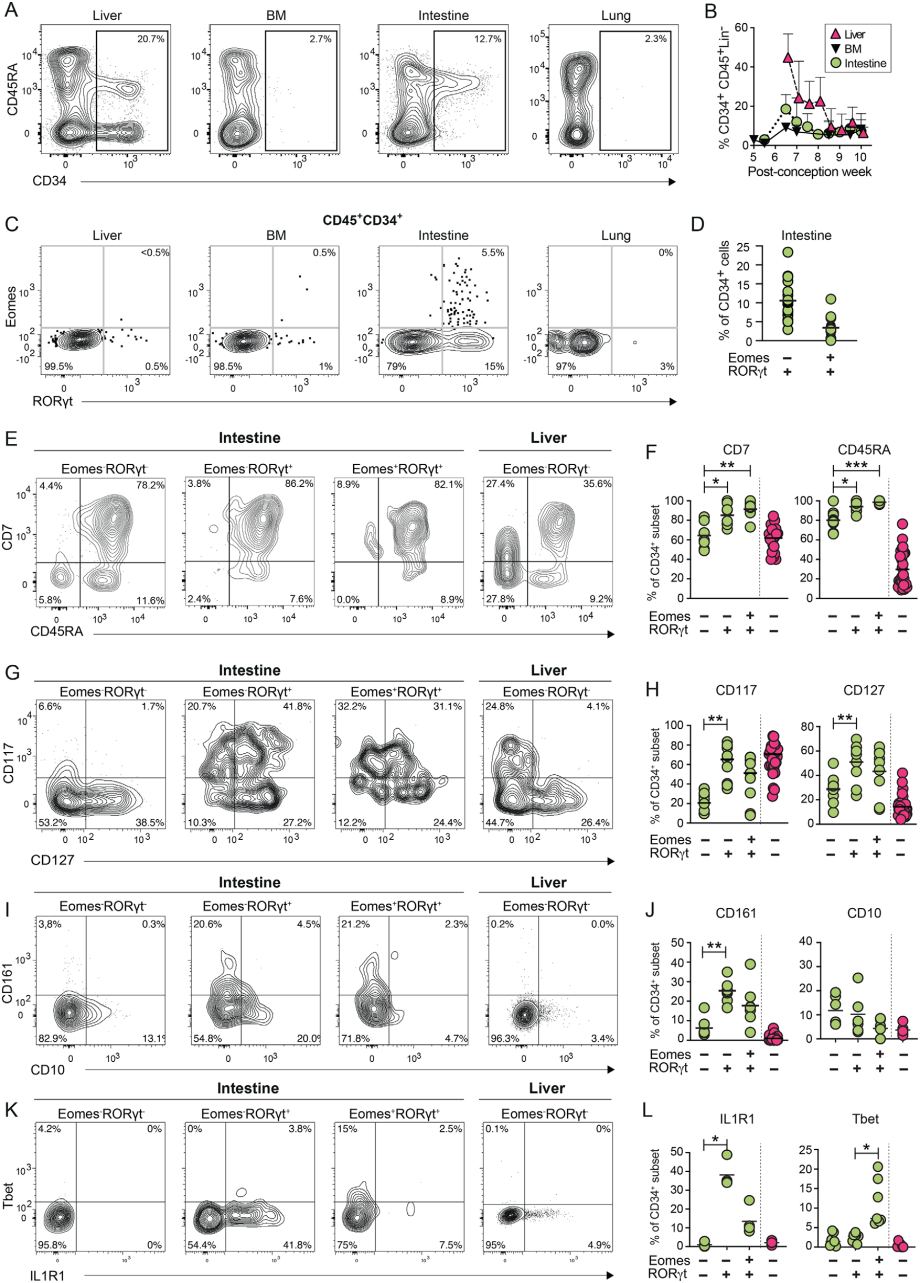
Fetal CD34^+^ cells co‐express Eomes and RORγt in the intestine. (A) Representative contour plots of CD45RA and CD34 expression by Lin^−^CD45^+^ cells in fetal liver, bone marrow, intestine, and lung at PCW 7.5. (B) Mean frequency of CD34^+^ cells of Lin^−^CD45^+^ cells across gestational age in fetal liver (*n* = 24), bone marrow (*n* = 13), and intestine (*n* = 24). (C) Representative contour plots of Eomes and RORγt expression by Lin^−^CD45^+^CD34^+^ cells in the liver, bone marrow, lung, and intestine at PCW 9.5. (D) Frequency of RORγt^+^Eomes^−^ and RORγt^+^Eomes^+^ of CD34^+^ cells in intestine (*n* = 17). Bar shows the mean frequency. (E–L) Representative contour plots (PCW 9.5) and summary of data for frequency of expression of (E, F) CD7 and CD45RA, (G, H) CD117 and CD127, (I, J) CD161 and CD10, and (K, L) Tbet and IL1R1 on CD34^+^RORγt^−^Eomes^−^, CD34^+^RORγt^+^Eomes^−^ and CD34^+^RORγt^+^Eomes^+^ cells in fetal intestine (green, *n* = 8–9), and on CD34^+^RORγt^−^Eomes^−^ cells in fetal liver (pink, *n* = 21–33), respectively. Statistical comparison by nonparametric ANOVA. *p_adj_ < 0.05, **p_adj_ < 0.01, ***p_adj_ < 0.001.

The frequencies of Lin^−^CD45^+^CD34^+^ cells were highest in the fetal liver and intestine, particularly early on in gestation, with substantially lower frequencies in BM and lung (Figure 4A,B). Within the Lin^−^CD45^+^CD34^+^ population, we identified CD34^+^RORγt^+^Eomes^−^ cells and CD34^+^RORγt^+^Eomes^+^ cells in the fetal intestine (Figure 4C,D), whereas they were scarce or absent in first‐trimester fetal liver, lung, and BM (Figure 4C). Virtually all CD34^+^RORγt^+^Eomes^−^ and CD34^+^RORγt^+^Eomes^+^ cells expressed CD45RA, and a large majority expressed CD7 (Figure 4E,F). Moreover, CD34^+^RORγt^+^Eomes^−^ cells more frequently expressed CD117, CD127, and CD161 compared with CD34^+^RORγt^−^Eomes^−^ cells, and a similar trend was observed for CD34^+^RORγt^+^Eomes^+^ cells (Figure 4G–J). CD10, reported to define NK cell‐restricted progenitors [28], was not more commonly expressed on CD34^+^RORγt^+^Eomes^−^ and CD34^+^RORγt^+^Eomes^+^ cells compared with CD34^+^RORγt^−^Eomes^−^ cells in the intestine (Figure 4I,J). Finally, CD34^+^RORγt^+^Eomes^−^ cells more frequently expressed IL1R1, a hallmark of ILC3s, whereas CD34^+^RORγt^+^Eomes^+^ more frequently expressed Tbet, compared with other CD34^+^ subsets (Figure 4K,L).

In summary, we identified CD45^+^CD34^+^ cells in the fetal intestine with hallmarks of NK cells and ILC3s, suggesting these cells could be tissue‐specific ILC progenitors.

### 2.4 Eomes and RORγt Are Co‐Expressed in CD34^−^CD16^–^ Fetal NK Cells

CD34^−^CD117^+^ ILC progenitors in adult tonsil and lung, cord blood, and fetal liver have been reported to develop into NK cells as well as other ILCs [13, 27, 31], indicating that the ILC subsets can diverge at CD34^−^ stages. In addition, adult CD16^−^ NK cells can express low levels of RORγt [29]. We, therefore, analyzed whether CD34^−^ fetal cells harbored cells with both NK cells and ILC3 characteristics and, if so, in which organs they reside.

**FIGURE 5:**
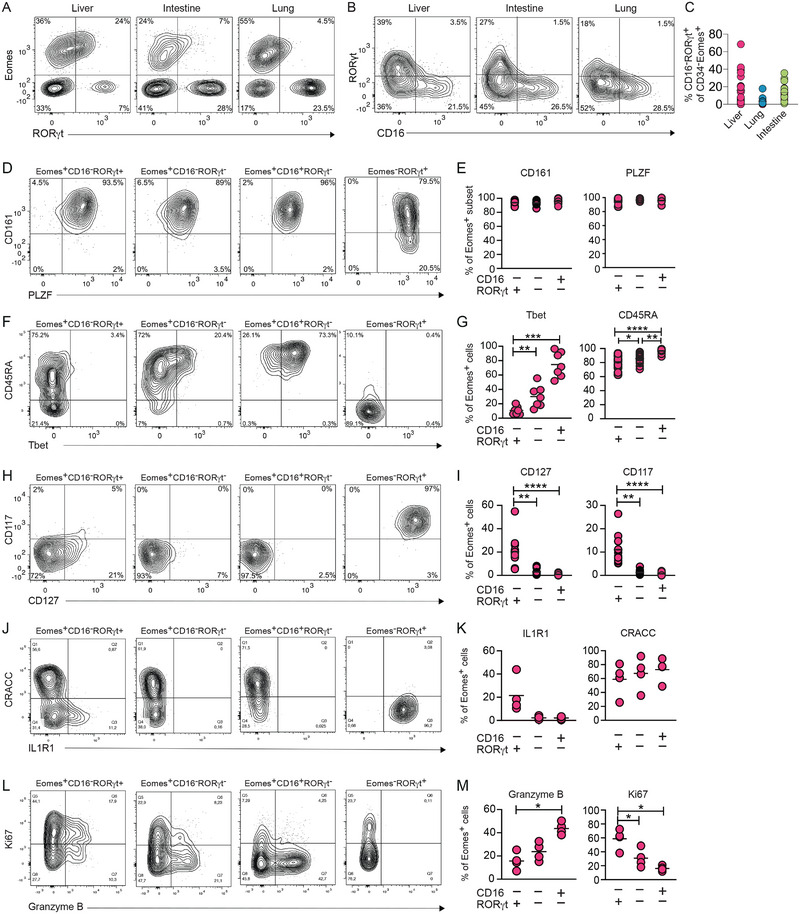
A subset of CD34^−^CD16^−^ cells co‐express RORγt and Eomes. (A) Representative contour plots for Eomes and RORγt expression by Lin^−^CD45^+^CD34^−^ cells in matched fetal liver, intestine, and lung at PCW 8. (B) Representative contour plots for RORγt and CD16 expression by Lin^−^CD45^+^CD34^−^Eomes^+^ cells in liver, intestine, and lung. (C) Frequency of CD16^−^RORγt^+^ of CD34^−^Eomes^+^ cells in liver (*n* = 23), lung (*n* = 16), and intestine (*n* = 17), PCW 7*p*_adj_20. (D–M) Representative contour plots and summary of frequencies of (D, E) CD161 (*n* = 15) and PLZF (*n* = 5), (F, G) Tbet (*n* = 7) and CD45RA (*n* = 15), (H, I) CD117 (*n* = 15) and CD127 (*n* = 15), (J, K) IL1R1 (*n* = 4) and CRACC (*n* = 5), and (L, M) Granzyme B (*n* = 5) and Ki67 (*n* = 5) on Eomes^+^CD16^−^RORγt^+^, Eomes^+^CD16^−^RORγt^−^, Eomes^+^CD16^+^RORγt^−^, and Eomes^−^RORγt^+^, respectively in fetal liver (PCW 7–12). Statistical comparison by nonparametric ANOVA. *p_adj_ < 0.05, **p_adj_ < 0.01, ***p_adj_ < 0.001, ****p_adj_ < 0.0001.

We identified Eomes^+^ NK cells expressing low levels of RORγt in the fetal liver, intestine, and lung (Figure 5A), and they were largely confined to CD16^−^ NK cells (Figure 5B,C). In contrast, RORγt^high^ ILC3s did not express Eomes (Figures 1A; Figure 5A). Next, we further characterized the Eomes^+^CD16^−^RORγt^+^ NK cells in the fetal liver by analyzing NK cells and ILC3‐related proteins. While all Eomes^+^ subsets expressed CD161 and PLZF (Figure 5D,E), the Eomes^+^CD16^−^RORγt^+^ cells more frequently expressed CD127, CD117, and IL1R1, and less frequently expressed Tbet, CD45RA, and granzyme B, compared with Eomes^+^CD16^−^RORγt^−^ cells and Eomes^+^CD16^+^RORγt^−^ cells (Figure 5F–K). Finally, Eomes^+^CD16^−^RORγt^+^ cells more frequently expressed Ki67 compared with Eomes^+^CD16^−^RORγt^−^ and Eomes^+^CD16^+^RORγt^−^ cells (Figure 5L,M).

Collectively, our data show that a subset of CD34^−^Eomes^+^ cells within fetal tissues express ILC3‐associated proteins, indicating that they could be derived from RORγt^+^ progenitors.

## 3 Discussion

Here, we demonstrated that mature human fetal ILCs are present in multiple tissues early during the first trimester of fetal development, with distinct subset composition, kinetics, and gene expression patterns between tissues. We further identified CD34^+^RORγt^+^Eomes^+^ and CD34^+^RORγt^+^Eomes^−^ cells exclusively in the fetal intestine, consistent with the notion of distinct tissue‐restricted ILC development, as has previously been suggested for adult ILCs [29, 30].

NK cells were the most frequent ILC population in the fetal liver, lung, and skin, and were present at a similar frequency as ILC3s in the fetal intestine. High expression levels of *IFNG*, *GZMA*, and *GZMB*, as well as CD16, already in the first trimester, indicate that fetal NK cells are indeed mature and functional, as previously reported for fetal NK cells in 2nd trimester tissues [18]. Fetal NK cells could therefore provide protection against infections, for example, via ADCC and maternally derived antibodies, or by production of IFNγ in response to proinflammatory cytokines. NK cell‐mediated protection against infections might be particularly important at the early stages of fetal immune development, where T cells are largely absent in the first trimester, and robust antibody responses by fetal B cells only emerge toward the end of the second trimester [42].

Despite the overall similar gene expression pattern in NK cells, we found tissue‐specific differences for CD16^−^ and CD16^+^ NK cells. For example, CD16^+^ NK cells in the lung, skin, and intestine expressed higher levels of KIRs, *FGFBP2*, and granzyme B compared with those in the liver, whereas CD16^+^ NK cells in the liver expressed higher levels of *IFNG*, *CD27*, and *GZMK*. In addition to their inhibitory function when binding HLA class I ligands, KIR expression by fetal NK cells has previously been shown to render them hyporesponsive [18]. The gene and protein expression profiles indicate that CD16^+^ NK cells in the lung, skin, and intestine are more differentiated and cytotoxic, and as such might require tighter regulation of cytotoxicity by KIRs. Conversely, CD16^+^ and CD16^−^ NK cells in the fetal liver expressed higher levels of *CXCR6* and *CRACC*. CXCR6 is also expressed at high levels by adult liver CD16^−^ NK cells [19, 43], and CXCR6^+^ NK cells have been suggested to be retained in liver sinusoids by CXCL16 produced by endothelial cells [43, 44] and Kupffer cells [17]. CRACC can also be upregulated on Kupffer cells by type I IFNs [45], potentially allowing CRACC–CRACC interactions between NK cells and Kupffer cells during viral infections. Taken together, this indicates that NK cells in the fetal liver are well suited to interact with liver endothelial cells and Kupffer cells, potentially playing a regulatory role via the production of IFN‐γ.

ILC3s with LTi function are important for the development of lymphoid structures in mice, including lymph nodes and Peyer's patches [46]. Consistent with an LTi‐like function, human fetal and adult ILC3s in lymphoid tissues express *LTA* and *LTB* and can induce the expression of ICAM and VCAM on mesenchymal stem cells [6, 21]. Fetal CD304^+^ ILC3s in all tissues expressed high levels of *LTA/B*, consistent with their proposed role as LTi cells [21]. In addition to their LTi‐like function, CD304^+^ ILC3s expressed *IL17A*, *IL17F*, *TNF*, and *CSF2*, suggesting that they could also be involved in regulating a controlled inflammatory milieu in the fetus.

Similar to NK cells, CD304^+^ ILC3s had distinct tissue‐specific gene expression patterns that likely contribute to differences in regulation and function. For example, fetal liver CD304^+^ ILC3s expressed higher levels of *PECAM1* (CD31) and *PTPN6* (SHP‐1). PECAM1 is an inhibitory receptor dependent on the phosphatase SHP‐1 for its inhibitory function and might mediate the inhibition of CD304^+^ ILC3s via interaction with PECAM1 expressed by endothelial cells in the liver [47]. Fetal liver CD304^+^ ILC3s also expressed lower RNA and protein levels of *IL2RA* (CD25) compared with lung and skin. Together with the higher expression of *PTPN6*, which also negatively regulates IL‐2 signaling, this likely results in poorer responses to IL‐2 by liver CD304^+^ ILC3s [48]. Additional tissue‐specific differences in CD304^+^ ILC3s included increased expression of activating receptors (*NCR2*, *CD2*) in the lung and intestine, indicating that they may be more prone to activation via cell–cell interactions. This has also been observed in the adult colon compared with blood and liver [26, 40], suggesting that this phenotype is maintained throughout development in the intestine. Furthermore, CD304^+^ ILC3s in the lung and intestine had increased expression of genes associated with tissue residency (*ALOX5AP*, *RGS1*, *RGS2*, and *ZNF331*) [22]. However, compared with CD304^−^ ILC3s, a lower frequency of CD304^+^ ILC3s expressed CD103, a hallmark of intraepithelial resident lymphocytes. Together this indicates that although both CD304^+^ and CD304^−^ ILC3s in the intestine and lung have a tissue‐resident profile, they may have distinct spatial localization and function. This is in line with previous results showing an enrichment of CD103^+^ ILC3s in second‐trimester fetal intestinal epithelium [20].

The observed tissue‐specific gene expression patterns may be due to imprinting by the local tissue environment, selective recruitment of mature ILCs, or tissue‐restricted ILC development. Tissue‐specific gene expression patterns were almost completely unique to each ILC subset and therefore argue against a universal tissue imprint on all immune cells in each tissue. Furthermore, selective recruitment of ILCs to different tissues remains a possible explanation for the tissue‐specific gene expression patterns, as exemplified by the enrichment of CXCR6^+^ NK cells in the liver. However, unlike NK cells, CD304^+^ ILC3s in the liver did not express CXCR6 more frequently, indicating that recruitment and/or retention of different ILCs to a tissue do not necessarily depend on the same chemokine receptor. The identification of CD34^+^RORγt^+^Eomes^+/−^ cells in the intestine, but not in other tissues, supports the notion of tissue‐specific ILC progenitors and development. However, direct evidence that the CD34^+^RORγt^+^Eomes^±^ cells in the fetal intestine indeed give rise to ILCs and NK cells is lacking. As such it also remains unknown whether these cells give rise to ILCs only in the intestine or if they possess the capacity to seed ILCs to other tissues, for example, the lung, as suggested for mouse ILC2s after pathogen challenge [49].

Previous studies have identified CD34^+^RORγt^+^ ILC‐restricted progenitors in adult secondary lymphoid tissues [29, 30], but not in peripheral blood, cord blood, thymus, or BM [29]. Downstream of the previously defined CD34^+^ ILC progenitors, CD34^−^ ILCPs in cord blood, adult blood, lung, tonsil, and fetal liver contain multipotent progenitors capable of differentiating into ILC1, ILC2, and ILC3s, suggesting a late diversification in ILC differentiation [13, 27]. In line with the phenotype of CILPs (CD34^+^CD45RA^+^CD117^+^IL‐1R1^+^CD127^±^ cells) in adult tonsil [29], a majority of the CD34^+^RORγt^+^Eomes^−^ cells in fetal intestine expressed CD117 and CD45RA, and a substantial fraction expressed CD127 and IL1R1. Overall, we gradually detected less proliferation and less expression of ILC3‐related markers (CD117, CD127, IL1R1), and more expression of NK cell‐related markers (Tbet, GZMB, CD94) going from CD34^+^RORγt^+^Eomes^−^ cells to CD34^+^RORγt^+^Eomes^+^ cells, and subsequently from CD34^−^RORγt^+^Eomes^+^CD94^+^CD16^−^ NK cells to CD34^−^RORγt^−^Eomes^+^CD94^+^CD16^−^ NK cells. Our data thus support the notion that at least some NK cells develop from a common CD34^+^ ILC progenitor in the fetal intestine, via a CD34^−^RORγt^+^Eomes^+^ NK cell stage. However, future single‐cell in vitro cloning experiments and ultimately in in vivo fate mapping experiments would be required to determine this.

### 3.1 Data Limitations and Perspective

We have identified putative CD34^+^RORγt^+^Eomes^+/−^ progenitors in the fetal intestine. We were, however, limited in further exploring the differentiation capacity of these cells in vitro, as fixation and permeabilization of the cells would be required to detect intracellular expression of Eomes and RORγt, precluding isolation of live cells.

In our analysis of tissue‐dependent gene expression, we sorted relatively homogenous ILC subpopulations. However, there is likely heterogeneity even within these subsets, which, in turn, limited our ability to determine whether preferential recruitment of subpopulations or imprinting by the tissue environment underlies the observed tissue‐dependent gene expression patterns we observe.

Future studies in a larger number of donors using single‐cell RNA sequencing and single‐cell in vitro differentiation assays could be helpful in further dissecting the tissue‐dependent transcriptional patterns and ILC developmental pathways.

## 4 Material and Methods

### 4.1 Human Tissues and Blood

First and second‐trimester fetal tissues were obtained from the Developmental Tissue Bank core facility at the Karolinska Institutet (PCW 6–12) and the Women's Options Center at San Francisco General Hospital (second trimester, gestational weeks 15–20), respectively. For first trimester samples, the postconceptional week was determined using ultrasound, anatomical landmarks, and actual crown‐rump‐length. For second‐trimester samples, postconceptional week was estimated by measuring footpad size. Tonsils were collected from adult patients (20–65 years of age) undergoing tonsillectomy because of obstructive sleep apnea syndrome.

To isolate cells, tissues were cut into smaller pieces and incubated for 30 min at 37°C with 0.25 mg/mL collagenase 2 (Sigma‐Aldrich), passed through a 70 µm nylon mesh, and diluted with R10 media (RPMI1640 with 10% FCS, penicillin, streptomycin, and L‐glutamine). For first‐trimester samples, 0.2 mg/mL DNase (Roche) was also added during the digestion. Isolated cells were either stained directly or cryopreserved in FCS with 10% DMSO and stored in liquid nitrogen until use. Fetal liver and adult tonsil samples used for the analysis of NKp80 expression (Figure S1C) were not digested using collagenase to avoid any potential effects of enzymatic activity.

### 4.2 Flow Cytometry

Antibodies and clones against the following proteins were used (clone, fluorophore, company): IL1R1 (FITC, polyclonal, R&D Systems), CD2 (RPA‐2.10, BUV395, BD Biosciences), CD3 (UCTH1, PE‐Cy5, Beckman Coulter), CD7 (Horizon V450 or BUV395, BD Biosciences), CD10 (HI10a, APC‐Cy7, Biolegend), CD14 (M5E2, Horizon V500 or PE‐Cy5, BD Biosciences, or BV510, Biolegend), CD16 (3G8, APC‐Cy7, BV570, or BUV496, BD Biosciences), CD19 (HIB19, Horizon V500 or PE‐Cy5, BD Biosciences, or BV510, Biolegend), CD25 (M‐A251, BV785, BD Biosciences), CD34 (581, ECD, Beckman Coulter, or PE‐Dazzle, Biolegend), CD45 (HI30, Alexa700, BioLegend, or BUV805, BD Biosciences), CD45RA (MEM‐56, Qdot655, Invitrogen, or HI100, BV785, Biolegend), CD94 (#131412, custom‐biotinylated, RnD Systems), CD94 (HP‐3D9, BB700, BD Biosciences), CD103 (Ber‐ACT8, BB660, BD Biosciences), CD117 (104D2D1, PE‐Cy5.5, Beckman Coulter or 104D2, BUV737, BD Biosciences), CD127 (R34.34, PE‐Cy7, Beckman Coulter, or A019D5, BV785, Biolegend), CD161 (HP‐3G10, Brilliant Violet 605 or PerCP, BioLegend), CD304 (12C2, BV421, Biolegend, or U21‐1283, BUV661, BD Biosciences), CRTH2 (BM16, Horizon V450, BD Biosciences), CXCR6 (KO41E5, BV421, BioLegend), CRACC (162.1, PE‐Cy7, BioLegend), Granzyme B (GB11, RB613, BD Biosciences), Ki67 (Ki‐67, BV750, BioLegend), KIR3DL1 (DX9 AlexaFluor700, BD Biosciences), KIR2DL1/S1 (EB6, Pe‐Cy5.5, Beckman Coulter), KIR2DL2/3/S2 (GL183, Pe‐Cy5.5, Beckman Coulter), NKp80 (4A4.D10, PE or PE‐Cy7, Miltenyi), Eomes (WF1928, FITC, or eF660, eBioscience), RORγt (AFKJS‐9, PE, eBioscience or Q21‐559, PE or BV650, BD Biosciences), GATA‐3 (TWAJ, eF660, eBioscience, or L50‐829, PE‐Cy7, BD Biosciences), T‐bet (4B10, BV421, Biolegend, or O4‐46, PE‐CF‐594, BD Biosciences), and PLZF (#6318100, APC, RnD Systems, or Mags.21F7, PE, eBioscience).

Secondary staining steps were performed using streptavidin Qdot585, Qdot605, Qdot 655 (Invitrogen), or streptavidin BV650 (Biolegend). All samples were stained with live/dead aqua or live/dead near‐IR (Invitrogen). After cell surface staining with antibodies diluted in FACS wash (PBS with 2% FCS and 2 mM EDTA), cells were fixed using a FoxP3 transcription factor staining kit (Invitrogen) for 30 min and incubated with antibodies against intracellular proteins for 30 min and resuspended in FACS wash before analyses using BD LSRII SORP, BD LSR Fortessa or BD Symphony flow cytometers. Data were analyzed using FlowJo v10 (BD Biosciences).

### 4.3 Statistical Analysis of Flow Cytometry Data

For matched samples with non‐Gaussian distribution, we used Friedman's test with Dunn's multiple comparisons test. For matched samples with Gaussian distribution, we used one‐way ANOVA with Geisser‐Greenhouse correction and Holm‐Sidak's multiple comparison test. Mixed‐effects analysis with Geisser‐greenhouse correction and Tukey's multiple comparison tests was used for matched samples with missing values. The significance of the correlation was done with the Pearson correlation test. Statistical analyses were performed using Prism software version 8.0 (GraphPad Software Inc).

### 4.4 Cell Sorting and RNA Sequencing

Thawed cryopreserved cells from fetal liver, lung, intestine and skin (*n* = 3, PCW 9.5) were stained with the following antibodies (clone, fluorophore, company): CD3 (UCHT1, FITC, Biolegend), CD7 (M‐T701, BUV395, BD Biosciences), CD14 (61D3, PE‐Cy5, Invitrogen), CD15 (W6D3, PE‐Cy5, Biolegend), CD16 (3G8, APC‐Cy7, BD Biosciences), CD19 (HIB19, PE‐Cy5, Biolegend), CD34 (581, ECD, Beckman), CD45 (HI30, AlexaFluor 700, Biolegend), CD94 (HP‐3D9, BB700, BD Biosciences), CD117 (104D2, BUV737, BD Biosciences), CD127 (HIL‐7R‐M21, BV421, BD Biosciences), CD304 (U21‐1283, BUV661, BD Biosciences), and live/dead aqua (Invitrogen) for 30 min at 4°C, followed by washing in FACS wash. NK cells and ILC3s were first enriched by gating on Live CD45^+^CD3^−^CD14^−^CD15^−^CD19^−^CD34^−^ cells and sorting CD94^+^ and CD94^−^CD127^+^ cells into separate tubes. From the enriched cells, three subsets were sorted: CD94^+^CD16^−^ NK cells and CD94^+^CD16^+^ NK cells from the CD94^+^ enriched cells and CD94^−^CD127^+^CD304^+^ ILCs from the CD94^−^CD127^+^ enriched cells (Figure S2A). For each subset from each organ 35–100 cells were sorted in duplicates into 4.2 µL of lysis buffer (0.2% Triton X‐100, 2.5 µM oligo‐dT [5′‐AAGCAGTGGTATCAACGCAGAGTACT30VN‐3′], 2.5 mM dNTP, RNAse Inhibitor [Takara]) in a 96‐well V‐bottom PCR plate (Thermo Fisher). Before further processing cells were stored at ‐80°C. RNA libraries were prepared using the standard SmartSeq2 protocol with 18 PCR cycles for cDNA amplification. The cDNA quality was assessed by a bioanalyzer (Agilent, High Sensitivity DNA chip). Two nanograms of amplified cDNA were used for our custom tagmentation protocol and indexed with Nextera XT primers. Samples were pooled and sequenced on a NextSeq2000 with a P3 100 flow cell.

### 4.5 Transcriptome Analysis

Following sequencing, base‐calling and demultiplexing were done using bsl2fastq (v2.20.0.422) with default settings generating Fastq files for further downstream mapping and analysis. Alignment was conducted using STAR 2.7.5b to GRC38/hg38.101 genome sequence from the ensemble. Count reads in exons were generated with featureCounts (v1.5.1) The resulting count matrix was analyzed using DESeq2 (v.1.38.3) in R (v.4.2.2). In short, raw counts from duplicates were averaged and used for input into DESEq2 and normalized. For each cell subset, only genes expressed by ≥2 samples with ≥50 counts were used for further analysis. Variance stabilizing transformation was used to transform the data, and limma (v.3.54.2) was used to correct donor batch effects. Differentially expressed genes were determined by adjusted *p*‐value < 0.01 and log2‐fold change >1 or <−1 by pairwise comparison between subsets (CD16^+^ NK cells vs. CD16^−^ NK cells, CD16^+^ NK cells vs. CD304^+^ ILC3s, and CD16^−^ NK cells vs. CD304^+^ ILC3s) and tissues (intestine vs. liver, lung vs. intestine, lung vs. liver, skin vs. intestine, skin vs. liver, and skin vs. lung). Z‐scores for differentially expressed genes were calculated and used for input in pheatmap (v.1.0.12) to generate heatmaps. For tissue‐specific differentially expressed genes, only genes uniquely differentially upregulated in one tissue for each subset were selected and visualized. Euler plots were generated using all differentially expressed genes and the nVennR (v.0.2.3) package. The adjusted *p*‐values from the differential gene expression analysis from the DESeq2 results were used to select genes of interest. For each subset, genes used for visualization (heatmaps, Euler plots, and gene expression plots) had to be present in at least two donors from the same tissue with counts greater than 200.

## Author Contributions

Inga E. Rødahl, Martin A. Ivarsson, and Jakob Michaëlsson designed the study, performed experiments, analyzed data, made figures, and wrote the manuscript. Liyen Loh collected and processed fetal tissues and provided technical expertise. Jeff E. Mold performed experiments. Danielle Friberg provided adult tonsil tissue. Jenny Mjösberg, Niklas K. Björkström, and Douglas F. Nixon provided input on the study design. Nicole Marquardt performed experiments, provided input on study design, and reviewed data. All authors provided input on the manuscript before submission.

## Ethics Statement

Tissue samples were collected after informed consent and with the approval of either the Swedish Ethical Review Authority or the University of California San Francisco (UCSF) Committee on Human Research.

## Conflicts of Interest

The authors declare no conflicts of interest.

## Supporting information



SUPPORTING INFORMATION

SUPPORTING INFORMATION

## Data Availability

RNA‐seq count matrices and metadata are available at Zenodo (https://doi.org/10.5281/zenodo.14202841).
