# Supplementary material for: Distinct Tissue‐Dependent Composition and Gene Expression of Human Fetal Innate Lymphoid Cells
Source: Eur J Immunol. 2024 Dec 15;55(2):e202451150. doi: 10.1002/eji.202451150 (PMC11830385; doi:10.1002/eji.202451150)
Supplement: Supplementary file 1 — SUPPORTING INFORMATION [file EJI-55-e202451150-s001.pdf]

Figure S1

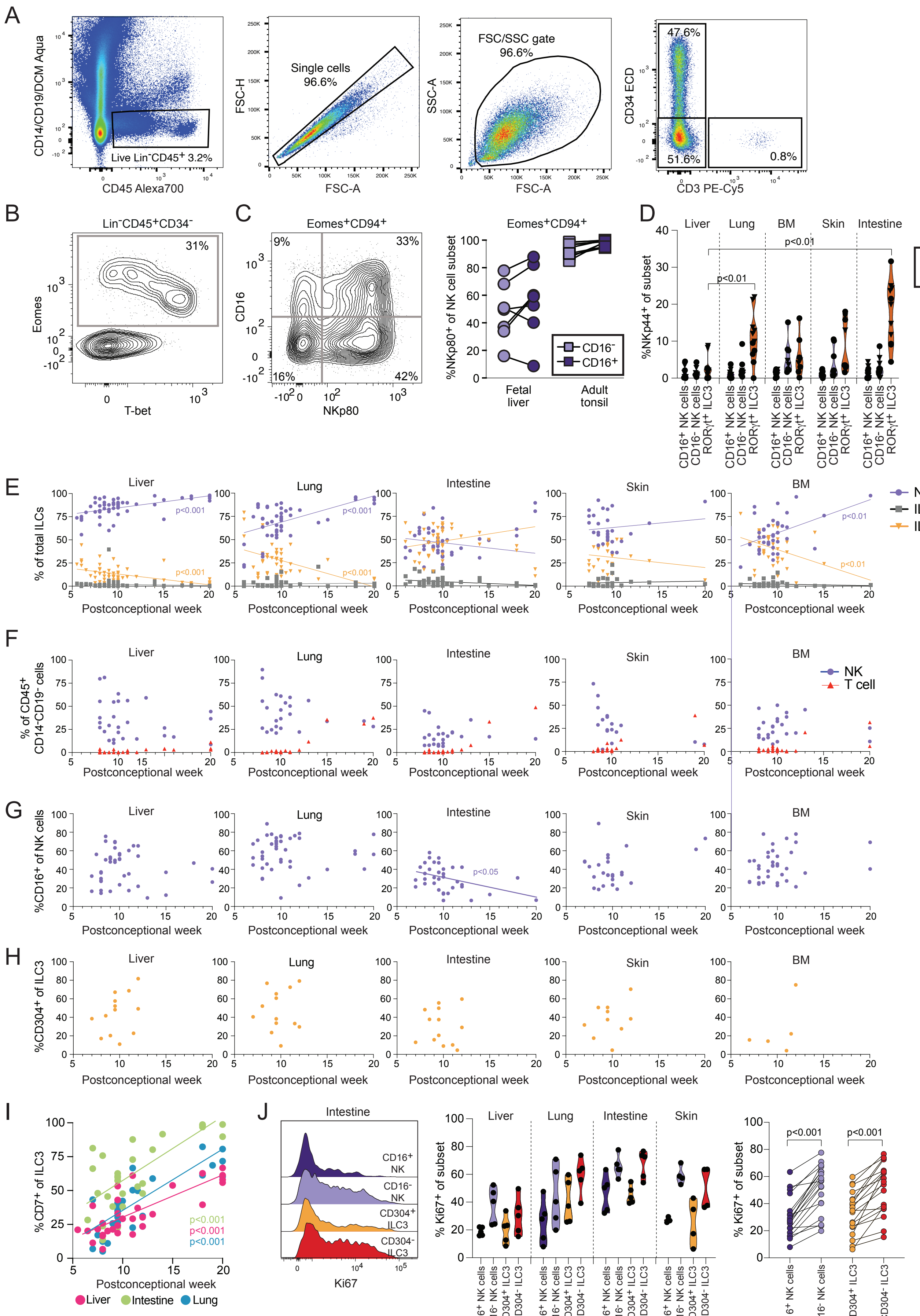

A
Live Lin⁻CD45⁺ 3.2%
CD14/CD19/DCM Aqua
CD45 Alexa700
Single cells 96.6%
FSC-H
FSC-A
FSC/SSC gate 96.6%
SSC-A
47.6%
51.6%
0.8%
CD34 ECD
CD3 PE-Cy5
B
Lin⁻CD45⁺CD34⁻
31%
Eomes
T-bet
C
Eomes⁺CD94⁺
9%
33%
16%
42%
CD16
NKp80
%NKp80⁺ of NK cell subset
CD16⁻
CD16⁺
Fetal liver
Adult tonsil
D
Liver
Lung
BM
Skin
Intestine
p<0.01
p<0.01
pcw >10
pcw <10
%NKp44⁺ of subset
CD16⁺ NK cells
CD16⁻ NK cells
RORγt⁺ ILC3
E
Liver
Lung
Intestine
Skin
BM
% of total ILCs
p<0.001
p<0.01
NK
ILC2
ILC3
Postconceptional week
F
% of CD45⁺ CD14⁻CD19⁻ cells
NK
T cell
G
%CD16⁺ of NK cells
p<0.05
H
%CD304⁺ of ILC3
I
% CD7⁺ of ILC3
p<0.001
Liver
Intestine
Lung
J
Intestine
CD16⁺ NK
CD16⁻ NK
CD304⁺ ILC3
CD304⁻ ILC3
Ki67
% Ki67⁺ of subset
CD304⁺ ILC3
CD304⁻ ILC3

Supplementary figure 1:

(**A**) Representative gating strategy for $CD45^{+}CD14^{-}CD19^{-}CD3^{-}CD34^{+}$ cells, $CD45^{+}CD14^{-}CD19^{-}CD3^{+}$ T cells and $CD45^{+}CD14^{-}CD19^{-}CD3^{-}CD34^{-}$ cells (PCW 8). (**B**) Representative contour plot of Eomes and Tbet expression in $Lin^{-}CD45^{+}CD34^{-}$ cells in fetal liver (PCW 8). (**C**) Representative contour plot of NKp80 and CD16 expression on fetal $Eomes^{+}CD94^{+}$ NK cells in fetal liver (left) and frequency of $NKp80^{+}$ cells of $CD16^{-}$ and $CD16^{+}$ NK cells in fetal liver (*n* = 7, PCW 7-14) and adult tonsil (*n* = 9) (right). (**D**) Frequency of $NKp44^{+}$ cells of $CD16^{+}$ and $CD16^{-}$ NK cells, and $ROR\gamma t^{+}$ ILC3 in fetal liver, lung, BM, skin, and intestine (*n* = 8-12). Statistical analysis by mixed-effects analysis with Geisser-greenhouse correction and Tukey's multiple comparison test. (**E**) Frequency of NK cells (purple), ILC2s (gray) and ILC3s (orange) of total ILCs over gestational age in fetal liver (*n* = 53), lung (*n* = 47), intestine (*n* = 47), bone marrow (BM, *n* = 44) and skin (*n* = 29). (**F**) Frequency of NK cells (purple) and T cells (red) of total $CD45^{+}CD14^{-}CD19^{-}$ cells across gestational age in fetal liver (*n* = 32), lung (*n* = 28), intestine (*n* = 26), BM (*n* = 27), and skin (*n* = 17). (**G**) Frequency of $CD16^{+}$ cells of NK cells over gestational age in fetal liver, lung, intestine, skin and BM. (**H**) Frequency of $CD304^{+}$ cells of ILC3s over gestational age in fetal liver, lung, intestine, skin and BM. (**I**) Frequency of $CD7^{+}$ cells among ILC3s over gestational age in fetal liver (pink, n= 31), intestine (green, n=28), and lung (blue, n=27). (**J**) Representative histogram (left), frequency across fetal liver, lung, intestine and skin (middle), and total frequency (right) of Ki67 expression by $CD16^{-}$ and $CD16^{+}$ NK cells, and $CD304^{-}$ and $CD304^{+}$ ILC3s (*n* = 5). Statistical analysis by mixed-effects analysis with Geisser-greenhouse correction and Tukey's multiple comparison test. Statistical significance of correlations was determined with Pearson correlation analysis.

Figure S2

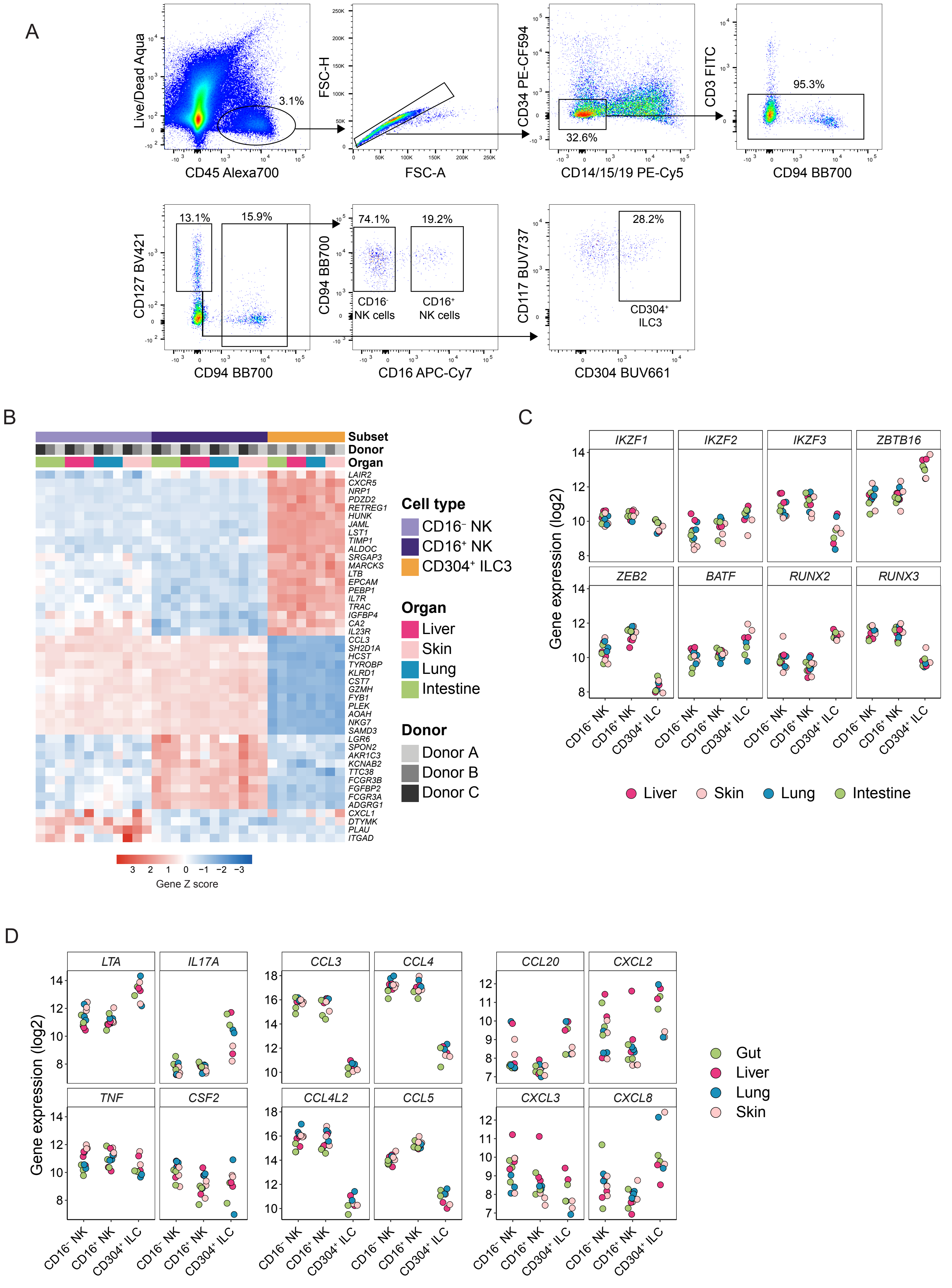
A
Live/Dead Aqua
CD45 Alexa700
3.1%
FSC-H
FSC-A
CD34 PE-CF594
CD14/15/19 PE-Cy5
32.6%
CD3 FITC
CD94 BB700
95.3%
CD127 BV421
13.1%
15.9%
CD94 BB700
74.1%
19.2%
CD16- NK cells
CD16+ NK cells
CD16 APC-Cy7
CD117 BUV737
28.2%
CD304+ ILC3
CD304 BUV661
B
Subset
Donor
Organ
LAIR2
CXCR5
NRP1
PDZD2
RETREG1
HUNK
JAML
LST1
TIMP1
ALDOC
SRGAP3
MARCKS
LTB
EPCAM
PEBP1
IL7R
TRAC
IGFBP4
CA2
IL23R
CCL3
SH2D1A
HCST
TYROBP
KLRD1
CST7
GZMH
FYB1
PLEK
AOAH
NKG7
SAMD3
LGR6
SPON2
AKR1C3
KCNAB2
TTC38
FCGR3B
FGFBP2
FCGR3A
ADGRG1
CXCL1
DTYMK
PLAU
ITGAD
Cell type
CD16- NK
CD16+ NK
CD304+ ILC3
Organ
Liver
Skin
Lung
Intestine
Donor
Donor A
Donor B
Donor C
3 2 1 0 −1 −2 −3
Gene Z score
C
IKZF1
IKZF2
IKZF3
ZBTB16
ZEB2
BATF
RUNX2
RUNX3
Gene expression (log2)
CD16- NK
CD16+ NK
CD304+ ILC
Liver
Skin
Lung
Intestine
D
LTA
IL17A
CCL3
CCL4
CCL20
CXCL2
TNF
CSF2
CCL4L2
CCL5
CXCL3
CXCL8
Gene expression (log2)
CD16- NK
CD16+ NK
CD304+ ILC
Gut
Liver
Lung
Skin

Supplementary figure 2:

(**A**) Gating strategy for FACS-sorting of $CD45^+CD14^-CD15^-CD19^-CD3^-CD34^-CD94^+CD16^+$, $CD45^+CD14^-CD15^-CD19^-CD3^-CD34^-CD94^+CD16^-$ NK cells, and $CD45^+CD14^-CD15^-CD19^-CD3^-CD34^-CD94^-CD127^+CD304^+$ ILC3s for RNA-seq. Representative plots from fetal intestine (PCW 9.5). (**B-D**) RNA expression analysis of $CD16^+$ and $CD16^-$ NK cells ($n = 3$), and $CD304^+$ ILC3s ($n = 2$) from fetal liver, skin, lung and intestine (PCW 9.5) (**B**) Pairwise differential gene expression comparing $CD16^-$ and $CD16^+$ NK cells and $CD304^+$ ILC3 in all tissues with heatmap showing z-score of top 10 most differentially expressed genes from each comparison. Bars annotate cell type, organ, and donor identity (by padj, log2 fold change > 1 or < −1, padj < 0.01), if one gene was differentially expressed in two comparisons it is only shown once. (**C**) Gene expression (log2) of transcription factors. (**D**) Gene expression (log2) of chemokines and cytokines.

Figure S3

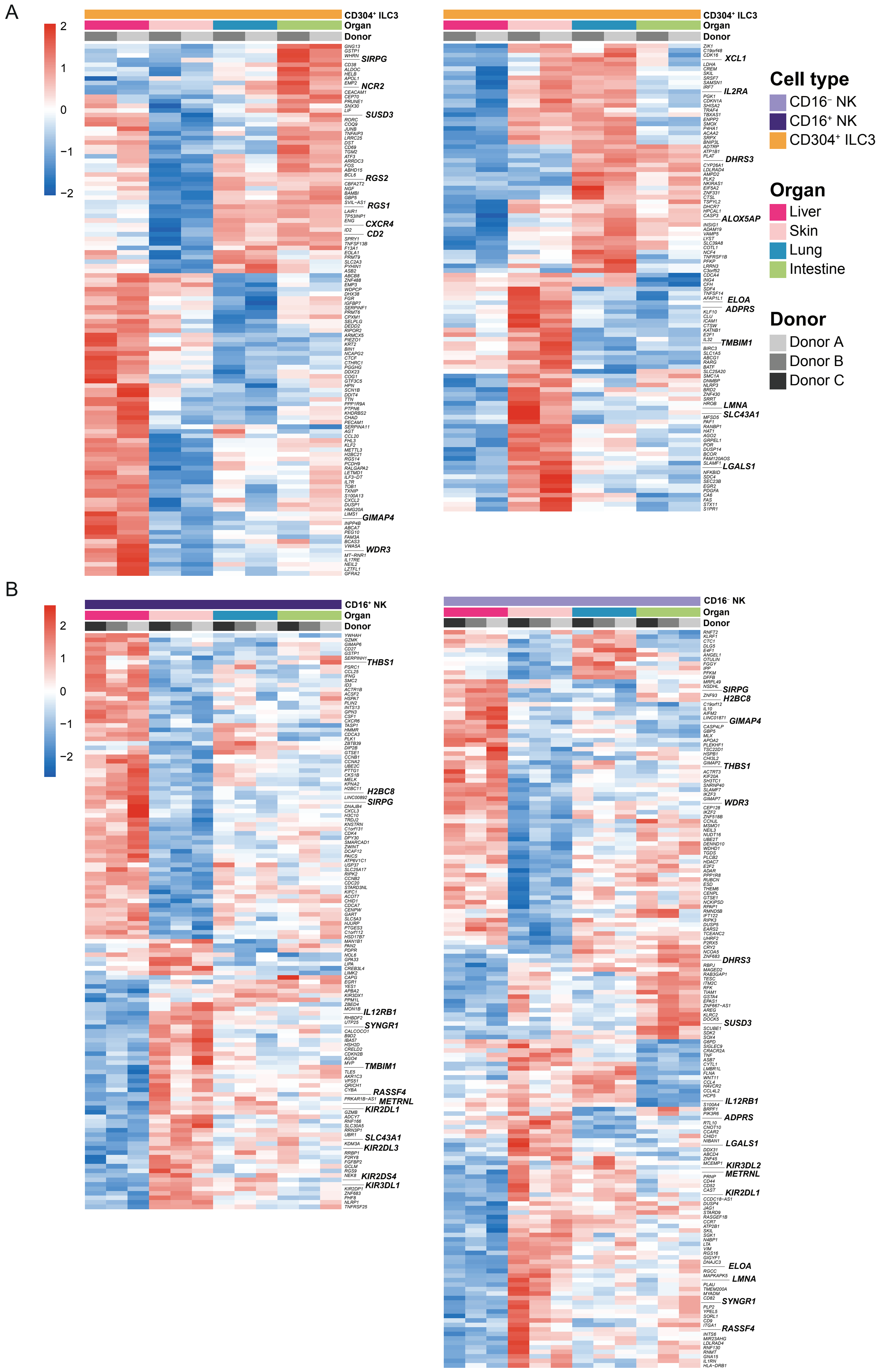

A
2
1
0
−1
−2
CD304⁺ ILC3
Organ
Donor
SIRPG
NCR2
SUSD3
RGS2
RGS1
CXCR4
CD2
GIMAP4
WDR3
XCL1
IL2RA
DHRS3
ALOX5AP
ELOA
ADPRS
TMBIM1
LMNA
SLC43A1
LGALS1
Cell type
CD16⁻ NK
CD16⁺ NK
CD304⁺ ILC3
Organ
Liver
Skin
Lung
Intestine
Donor
Donor A
Donor B
Donor C
B
CD16⁺ NK
THBS1
H2BC8
SIRPG
IL12RB1
SYNGR1
TMBIM1
RASSF4
METRNL
KIR2DL1
SLC43A1
KIR2DL3
KIR2DS4
KIR3DL1
CD16⁻ NK
SIRPG
H2BC8
GIMAP4
THBS1
WDR3
DHRS3
SUSD3
IL12RB1
ADPRS
LGALS1
KIR3DL2
METRNL
KIR2DL1
ELOA
LMNA
SYNGR1
RASSF4

Supplementary figure 3:

(**A-B**) Heatmaps showing z-scores of all differentially expressed genes, clustered hierarchical by row, from pairwise comparison between fetal tissues for (**A**) $CD304^+$ ILC3 (orange, $n = 2$), and (**B**) $CD16^-$ NK cells (light purple, $n = 3$) and $CD16^+$ NK cells (dark purple, $n = 3$). Bars annotate cell type, organ, and donor identity (padj<0.01, log2 fold change>1). If one gene was differentially expressed in two comparisons within one subset, only shown once.

Supplementary table 1 Genes uniquely upregulated by one tissue

Green = compared to lung, orange = compared to gut, blue = compared to liver, purple = compared to skin

$CD304^{+}$ ILC3 Organ	Top5 genes uniquely upregulated by one tissue from each comparison			Membrane immune regulatory	Secreted	Transcription factors or repressors	Other membrane	Other
Liver	NCAPG2<br>PTPN6<br>COG1<br>TTN<br>DEDD2	PEG10<br>LZTFL1<br>H2BC21<br>MT-RNR1<br>TTN	NCAPG2<br>SERPINA11<br>PIEZO1	IL7R<br>IL17RE	CCL20<br>CXCL2<br>IGFBP7<br>FAM3A	ZNF488<br>KLF2<br>CTCF	PECAM1<br>SLPLG<br>PIEZO1<br>SCN1B<br>PCDH9	PTPN6<br>RIPOR2<br>TXNIP<br>GIMAP4<br>BCAS3
Skin	EGR2<br>LGALS1<br>LMNA<br>ICAM1<br>AGO2	ICAM1<br>ADPRS<br>SDC4<br>STX11<br>LGALS1	ICAM1<br>MFSD5<br>IL32<br>TMBIM1<br>ELOA	SLAMF1<br>RARG	IL32<br>PDGFA<br>CFH<br>TNFSF14	BCOR<br>ZNF430<br>KLF10<br>BATF<br>E2F1	EGR2<br>LGALS1<br>ICAM1<br>SDC4<br>SEC23S1PR1	STX11<br>BIRC3
Gut	EMP2<br>GSTP1<br>SIRPG<br>CEACAM1<br>APOL1	GSTP1<br>ATF3<br>GNG13<br>CD38<br>GBP5		SIRPG<br>CD38	APOL1<br>TNFSF13B<br>LIF	ATF3	CEACAM1<br>CD69	
Lung	XCL1<br>ZNF331<br>LRRN3<br>CTSL1<br>LDHA	SLC2A3<br>AMPD2<br>ZNF331<br>PRMT9<br>PYHIN1			XCL1	ZNF331	LRRN3<br>TNFRSF1B	CTSL1<br>LYST

$CD16^{+}$ NK cells Organ	Top5 genes uniquely upregulated by one tissue from each comparison			Membrane immune regulatory	Secreted	Transcription factors or repressors	Other membrane	Other
Liver	CD27<br>SIRPG<br>GIMAP6<br>GSTP1<br>LINC00892	SIRPG<br>CSF1<br>H2BC11<br>KNSTRN<br>CXCL3	CCNB1<br>PLK1<br>H2BC11<br>YWHAH<br>NOL6	CD27<br>SIRPG	GZMK<br>IFNG<br>CXCL3<br>CSF1<br>CCL25<br>THBS1	ID3	CXCR6<br>HMMR	RIPK2<br>PLIN2<br>CMK4
Skin	RHBDF2<br>IBA57<br>HSH2D<br>UTP25<br>IL12RB1	CREB3L4<br>GPA33<br>LIMK2	PDPR<br>MAN1B1<br>LIPA	IL12RB<br>TNFRSF25	GZMB<br>FGFBP2	ZNF683	GPA33	RHBDF2<br>SYNGR1<br>MON1B<br>CYBA<br>RGS9<br>LIPA
Gut	CAPG<br>YES1<br>EGR1	CAPG	CAPG			EGR1		
Lung	KIR3DX1<br>PPM1L<br>APBA2	ZBTB39<br>DIP2B<br>USP37<br>GTSE1						PPM1L

CD16[illegible] NK cells Organ	Top5 genes uniquely upregulated by one tissue from each comparison			Membrane immune regulatory	Secreted	Transcription factors or repressors	Other membrane	Other
Liver	APOA2<br>MRPL49<br>CHI3L2<br>H2BC8<br>AIFM2	APOA2<br>H2BC8<br>IKZF3<br>IKZF2<br>WDR3	GBP5<br>APOA2<br>MLX<br>IL10<br>GIMAP4	SLAMF7<br>SIRPG	APOA2<br>CHI3L2<br>IL10<br>THBS1	IKZF2<br>IKZF3<br>TSC22D1		WDR3<br>MSMO1<br>GIMAP4<br>DENND10<br>GBP5
Skin	PLAU<br>RGS16<br>TMEM200A<br>LTA<br>YPEL5	CRACR2A<br>TNF<br>S100A4<br>ABCD4<br>DDX31	RTL10<br>CD9<br>CCAR2<br>PIK3R6<br>ADPRS	CD52	LTA<br>TNF<br>METRNL<br>IL1RN<br>PLAU		CD9<br>CD44<br>PRNP<br>HLA-DRB1	N4BP1<br>LGALS1<br>SYNGR1<br>CHID1<br>VIM
Gut	SCUBE1<br>KLRC2<br>DHRS3<br>TESC<br>SDK2	CCNJL<br>NCOA5<br>RMND5B<br>RPAP1<br>SCUBE1	ITGA1<br>SUSD3<br>BRPF1<br>TESC	KLRC2		RBPJ<br>SOX4	TIAM1<br>AREG<br>ITGA1	DHRS3<br>TESC
Lung	WNT11<br>MAGED2	DFFB<br>DLG5<br>IPP<br>E4F1<br>FGGY	CCL4L2<br>DLG5<br>FLNA<br>WNT11<br>KLRF1	KLRF1	WNT11<br>CCL4L2<br>CCL4	E4F1		
