# Supplementary material for: Distinct Tissue‐Dependent Composition and Gene Expression of Human Fetal Innate Lymphoid Cells
Source: Eur J Immunol. 2024 Dec 15;55(2):e202451150. doi: 10.1002/eji.202451150 (PMC11830385; doi:10.1002/eji.202451150)
Supplement: Supplementary file 2 — SUPPORTING INFORMATION [file EJI-55-e202451150-s002.pdf]

CD16^{-} NK cells differentially expressed genes padj < 0.05 log2foldchange > 1 or < –1

INTESTINE VS LIVER

Genes	baseMean	log2FoldChange	padj
ITM2C	5256,17679	2,793994816	7,94833E-18
EPAS1	1129,941838	4,849845502	6,15066E-14
GBP5	2535,274281	-1,512635437	1,13136E-08
ZNF683	894,9726842	4,539401477	2,9294E-08
SCUBE1	82,43037415	9,500029408	5,79165E-06
JAG1	746,1374312	2,517720646	5,7993E-06
KLRC2	764,5546619	1,975171017	7,66019E-06
CD82	452,4596433	3,854497527	1,68924E-05
DHRS3	9522,670054	1,100873418	2,45471E-05
IL12RB1	372,8533885	-2,130671533	8,06165E-05
TESC	630,6555041	2,567924325	8,46427E-05
APOA2	118,3698653	-5,610158169	0,000120739
DUSP4	1450,159581	2,67880166	0,000120739
MLX	769,4699839	-1,247744054	0,000454
SDK2	103,1872397	6,193526004	0,000561097
IL10	93,89144809	-6,536030427	0,000690698
CRY2	168,0976359	2,447177684	0,000806414
ZNF667-AS1	364,9458946	2,333457343	0,001271825
RBPJ	1170,698222	1,388345522	0,001449489
GSTA4	169,0855816	4,017378467	0,001449489
GIMAP4	3468,212716	-1,215049828	0,001449489
CASP4LP	455,9018795	-1,659651277	0,001449489
TIAM1	228,9695938	2,428207721	0,001784416
G6PD	1319,56167	-1,393996831	0,00204711
AREG	3478,764943	2,063455674	0,002332046
SOX4	1213,079402	1,56837676	0,00253149
RFK	236,9415458	2,061273166	0,002753833
CTC1	468,5457183	-1,540716361	0,002815854
RASGEF1B	2634,487643	1,305358342	0,00357383
KIR2DL1	377,7665366	3,866246153	0,004171418
AIFM2	68,9606045	-4,736038832	0,006407968
RNFT2	105,584141	-5,275714051	0,006407968
LMNA	2802,190632	2,216534434	0,006932179
RAB3GAP1	176,4971717	3,146967936	0,006932179
SIGLEC9	285,4666448	-2,496539751	0,007320881
STARD9	135,0430084	2,904061035	0,007952221
C19orf12	187,2165328	-2,516840106	0,008255256
LINC01871	723,9075629	-1,374445807	0,009551791
RNF130	364,3769283	2,806526751	0,009669566
DOCK5	112,4487325	3,275593441	0,009669566
PLEKHF1	165,6599844	-2,877793279	0,009850855
ARHGAP19	208,4236411	-2,546751497	0,010552097
GRN	483,6874285	-2,786072072	0,010552097
SUSD3	204,1082751	3,396261887	0,012412746
IL1RN	262,958137	2,942298874	0,017564338
LINC00861	4479,466458	-1,227243839	0,019044815
SLC25A51	185,0751556	2,644915733	0,019249782
DLG5	230,1071067	-1,838606313	0,023765244
PALLD	248,2860465	2,402501826	0,024794344
PTAFR	148,4176891	8,039327038	0,025072086
TMEM200A	129,774722	3,566681166	0,02561897
PLK2	420,6859157	1,869848041	0,025687148
PXMP4	226,3120217	-2,491663916	0,025687148
SYNGR1	491,7914392	1,640827135	0,025687148
ITGA1	157,3703265	3,923552152	0,027451597
ARL4A	790,9905185	1,123910457	0,027493906
PLAU	479,0783111	2,918706828	0,027493906
RGCC	384,1333302	1,566063401	0,029408577
PHF1	319,110529	1,517883295	0,029867124
TCF3	192,9870554	2,454292923	0,030151614
AMPD2	518,7792067	1,988004814	0,030483644

CSPP1	190,0478425	2,046428073	0,030483644
KLHL6	514,2983263	2,138817243	0,031727475
AFDN	148,9165076	-1,964640655	0,032445709
CYB561D2	1073,690614	-1,159758604	0,033339311
CNTROB	190,7243245	-3,07915538	0,034328682
ARHGEF39	367,8166716	-1,659142486	0,034616946
SLC5A6	688,6287261	1,924179687	0,035280563
TGFA	135,3189714	2,897798649	0,035280563
ENTPD5	160,4756739	3,069043332	0,036084306
LNX1	154,8231442	3,802364035	0,039588468
YPEL5	6148,880101	1,255219827	0,04151227
GPR183	4107,504299	1,546265149	0,04151227
GCNT1	256,8631073	3,410372818	0,049316646
USP2	123,4190536	3,901482931	0,049316646
CD9	351,1764438	3,129545799	0,049316646
ADAM12	572,1338746	-1,89120322	0,049680142

LUNG VS INTESTINE

**Genes**	**baseMean**	**log2FoldChange**	**padj**
IL12RB1	372,853388	2,66477788	2,9174E-07
CCL4L2	75415,4354	1,58471116	2,8247E-05
EPAS1	1129,94184	-3,1169464	4,4864E-05
ITM2C	5256,17679	-1,6222818	4,4864E-05
HAVCR2	3734,99974	1,54943617	4,4864E-05
ITGA1	157,370327	-6,2987136	0,00012274
DLG5	230,107107	2,59417659	0,00013233
SUSD3	204,108275	-4,6187741	0,00014167
FLNA	2499,30723	1,30167277	0,00019128
BRPF1	133,984297	-3,4889025	0,00139943
LMBR1L	170,15272	3,62378282	0,00143579
CTC1	468,545718	1,63591363	0,00195817
WNT11	164,747057	3,38481748	0,00371396
KLRF1	1729,6822	1,00918207	0,00447011
HCP5	679,316176	1,87057663	0,00475777
E4F1	256,425702	3,52767763	0,00575333
CYTL1	276,562554	3,13376556	0,00724661
TESC	630,655504	-2,0718	0,0073536
CCL4	186609,704	1,12137393	0,00984882
UBE2Q1	140,724377	-2,5630307	0,01452924
ISYNA1	994,163754	-1,6665467	0,01452924
MTSS1	602,036153	1,45826551	0,0201603
TASP1	458,749092	-2,4485129	0,0201603
SMIM24	166,055277	-2,8332475	0,0201603
RFLNB	174,218677	-2,7390313	0,02627623
KIR3DL2	227,730153	3,72110257	0,03276709
RASGRP2	379,959107	1,53014615	0,03328106
OPTN	1410,19125	1,05922081	0,03695623
TRMT1	719,986675	1,61870797	0,03695623
ZNF93	124,059359	-3,103755	0,03695623
ULBP2	387,040331	1,99143089	0,0377985
HGS	573,285087	1,98573284	0,0377985
TMEM50B	444,479305	1,5223653	0,0377985
ZNF71	142,986014	-3,9579918	0,03831563
RNFT2	105,584141	4,59570373	0,04282495
KLRC2	764,554662	-1,2258845	0,04754995

LUNG VS LIVER

**Genes**	**baseMean**	**log2FoldChange**	**padj**
CCR7	2643,10572	2,82895524	2,0719E-05
KIR2DL1	377,766537	5,2322814	2,0719E-05
APOA2	118,369865	-6,4025339	3,4788E-05
RASGEF1B	2634,48764	1,66754689	4,9972E-05
WNT11	164,747057	4,04156153	0,00012135
JAG1	746,137431	2,28480133	0,00012135

MRPL49	817,511745	-1,4911892	0,00049343
ZNF93	124,059359	-4,1752643	0,00049343
ZNF683	894,972684	3,29946401	0,00078137
CHI3L2	170,350447	-3,7074582	0,00105594
H2BC8	413,692085	-2,4573777	0,00105594
AIFM2	68,9606045	-5,5444622	0,00105711
MAGED2	2795,0251	1,01899269	0,00154086
NSDHL	296,747811	-2,5619259	0,00154086
CD82	452,459643	3,19507372	0,00154086
KIR3DL2	227,730153	4,63051914	0,00154086
DUSP4	1450,15958	2,22794859	0,00727688
CYTL1	276,562554	3,05308533	0,01074908
UCK1	223,167014	-3,4782142	0,01074908
COQ9	699,437527	-1,4568275	0,01091479
CLIC3	6633,60304	1,0528193	0,01216529
PLAU	479,078311	3,29671372	0,01234939
METRNL	1418,8268	1,51746425	0,01234939
MCEMP1	351,882662	3,49823073	0,01247456
KIR2DL3	341,579888	2,8123425	0,01251907
E4F1	256,425702	3,30589032	0,01267814
KRT23	163,217131	5,45648602	0,01400172
CCDC18-AS1	698,987206	1,73855947	0,01439725
NT5DC3	154,870369	-4,4422942	0,01439725
ITM2C	5256,17679	1,17171297	0,01565965
CXCL3	667,964309	-2,8594657	0,01724671
MAP2K3	1982,36933	1,62992363	0,01724671
PRNP	1320,03293	1,16616029	0,01861401
LMNA	2802,19063	2,08408165	0,01965249
LTA	3108,29769	1,20858333	0,0206068
KIR2DS4	261,918935	3,9726015	0,02110342
TASP1	458,749092	-2,3834479	0,02266256
ATP2B1	1788,24329	1,09186893	0,02327107
PSMC3IP	221,269669	-1,9321285	0,02601793
SCUBE1	82,4303741	6,09667735	0,02676768
ZNF45	214,939648	3,93251501	0,02782801
GRK6	823,691055	-1,1965426	0,0286769
TBCCD1	535,623753	-1,5805547	0,02896102
LINC00892	1321,39733	-1,5971925	0,0291911
RGS16	2031,69361	1,75301372	0,03019951
NFE2L2	5270,17821	1,12089015	0,03019951
CD83	14487,9274	1,2956291	0,03019951
BRPF1	133,984297	-2,6823049	0,03152461
STIP1	2581,02166	-1,0126441	0,03152461
TCF3	192,987055	2,49808158	0,03152461
UBE2F	533,832472	1,0173959	0,03154098
LINC01871	723,907563	-1,2352511	0,03604038
SIRPG	953,98196	-2,0974809	0,03604038
IL17C	136,358095	4,37178273	0,04131392
EPHA4	497,197234	1,9927488	0,04166221
SATB1	2544,90678	1,29714687	0,0429789
N4BP1	285,364395	1,67122694	0,0429789
DNAJB4	407,655913	-1,8707406	0,0436972
APC	200,746075	-1,9881249	0,04465979
HSPB1	1874,52403	-1,7939672	0,04465979
SALL4	246,793088	2,77959205	0,04566109
CCAR2	352,160582	-1,4153081	0,04573096
RBPJ	1170,69822	1,05777109	0,04752215
ARIH1	385,185983	1,38721385	0,04949822

SKIN VS INTESTINE

**Genes**	**baseMean**	**log2FoldChange**	**padj**
IL12RB1	372,853389	2,42248329	1,5992E-05
CRY2	168,097636	-3,0367349	4,1118E-05
CYTL1	276,562554	3,81037687	0,0002557

UHRF2	311,706829	-1,9416832	0,0002557
CRACR2A	483,249854	2,31324631	0,0002557
SIGLEC9	285,466645	3,16195631	0,0002557
TNF	2218,03605	1,93139095	0,00026884
E2F2	208,363193	-3,7588284	0,00032758
CCNJL	117,400963	-4,9650987	0,00048573
ZNF93	124,059359	-4,3359811	0,00088696
TCEANC2	174,169466	-3,2685548	0,00148678
S100A4	6610,19901	1,0710576	0,00148678
RUBCN	170,065661	-2,84155	0,00148678
ABCD4	826,105107	1,56211277	0,00162002
G6PD	1319,56167	1,41857037	0,00180693
ADAR	1136,84076	-1,398554	0,00186407
NCOA5	208,840588	-3,3364633	0,00314096
RMND5B	90,112723	-4,9720984	0,00323279
DDX31	189,751674	2,32351293	0,00391852
RPAP1	213,099356	-3,0511006	0,00429774
ANGEL1	168,317653	-3,3709044	0,00437725
HAVCR2	3734,99974	1,19875656	0,00457484
DUSP5	1273,78239	-1,2266168	0,00468012
ASB7	195,460245	2,04387114	0,00549657
PLCB2	438,046477	-2,1494343	0,00644343
ITM2C	5256,17679	-1,2217848	0,00646674
LMBR1L	170,15272	3,18561291	0,00657123
HCP5	679,316176	1,74493632	0,00838159
SCUBE1	82,4303742	-5,5849594	0,00838159
IFT122	301,840523	-2,9571459	0,00996202
DCP1B	99,6173496	-3,8888851	0,01052112
STX4	370,641433	1,66278545	0,01081981
ZNF136	192,977021	-3,0263714	0,01081981
TTLL4	165,140874	-4,1237266	0,01094668
CCL4L2	75415,4354	1,08938059	0,0111042
LZTFL1	749,5271	-1,1842428	0,01111518
TPK1	223,186353	2,61140403	0,01111518
NUDT16	145,290685	-3,1515388	0,01111637
MSMO1	364,464322	-1,6880761	0,01111637
CD27	684,685395	1,44167021	0,01111637
IPP	144,180438	-2,5156619	0,01206961
TNFRSF25	593,844816	1,62637823	0,01206961
H2BC8	413,692085	-2,0337246	0,01206961
ATP6V0E2	291,815886	1,90405481	0,01206961
DENND10	216,890031	-2,003371	0,01206961
ESD	879,289072	-1,1292346	0,01269868
CYB561D2	1073,69061	1,25868783	0,01368843
PFKM	103,429606	-3,1177274	0,01406814
ZNF683	894,972684	-2,5774515	0,01446433
ADGRG5	483,187064	2,42462987	0,01446433
TGDS	276,383863	-1,6594695	0,01452588
NCKIPSD	118,976609	-3,5196851	0,01475096
ELAC2	1158,57755	1,2523934	0,01549024
MYO19	361,660912	-2,191252	0,01866541
P2RX5	432,957102	-1,4452785	0,02080556
PHF8	216,116322	-2,6178523	0,02115757
ZNF346	355,450518	-1,7392821	0,02187713
NEU1	973,873935	1,40883953	0,02191659
DFFB	86,4084483	-2,7595857	0,02258668
ELK3	346,931974	1,30643818	0,02258668
WDHD1	484,378954	-1,3427332	0,02273339
THEM6	219,656732	-2,5893535	0,02576429
PLK3	223,007232	3,43481199	0,02657297
AFF3	288,342983	-2,1532033	0,02657297
LAT2	2727,76411	1,14111418	0,02657297
ULBP2	387,040331	1,94534592	0,02657297
EHMT2	189,474248	-2,2135162	0,02657297

LTA	3108,29769	1,12257891	0,02657297
IRF4	1185,12202	-1,8050516	0,02721417
SNX20	781,611305	1,34696202	0,02731678
MEI1	1287,46534	1,40506697	0,02784924
NIBAN1	372,166095	1,54139698	0,02887038
PPP1R8	88,4644879	-3,6216117	0,02912895
PTAFR	148,417689	-7,7756168	0,02912895
HILPDA	169,445018	-3,1355811	0,02912895
RCBTB2	567,190949	-2,3293547	0,02912895
ARHGAP19	208,423641	2,26443437	0,02967594
ZNF518B	191,074086	-2,4479252	0,02988287
CENPL	304,041503	-2,6067291	0,03008683
MTSS1	602,036153	1,31098847	0,03008683
PCTP	171,319722	-3,1427794	0,03020389
ATXN7L3B	613,09693	-1,3923029	0,03115569
ABCG1	696,036381	1,69647928	0,03115569
BEX3	682,199739	-1,5056325	0,03210077
HDAC7	388,859688	-1,4094717	0,03295808
CDK5RAP2	365,798918	-2,4521279	0,0348657
ING1	501,894974	1,49683918	0,0348657
JPT2	119,92169	-3,1409046	0,04051767
LPGAT1	218,450531	-1,9224044	0,04107768
ZBP1	298,845843	2,02388997	0,04285399
RIPK3	113,136687	-2,5914396	0,043743
B9D2	276,871358	1,86976455	0,043743
NR1H2	1435,02569	1,80409755	0,043743
ILVBL	255,722865	2,29379717	0,04583057
IDS	402,86382	1,56316214	0,0460577
C9orf64	108,211868	-3,3712371	0,04696975
VPS13D	267,987706	-2,0486947	0,04706378
TMPRSS3	126,512669	-3,3603139	0,04706378
ADAM12	572,133875	1,83810323	0,04855526
EPAS1	1129,94184	-1,853159	0,04894881
UBE2T	893,99532	-2,0700706	0,04906667
DUSP22	354,500068	2,00025223	0,04945104
TMEM131	524,462491	1,43784595	0,04987948
MEIS1	278,588745	1,40758905	0,04997758
PTPRK	131,577929	-2,7644756	0,04997758

SKIN VS LIVER

Genes	baseMean	log2FoldChange	padj
LMNA	2802,19063	3,96525724	8,1884E-10
PLAU	479,078311	5,00287481	9,1447E-07
DUSP5	1273,78239	-1,8081311	9,1447E-07
ZNF93	124,059359	-5,4074903	1,9921E-06
APOA2	118,369865	-6,8697174	5,2847E-06
RGS16	2031,69361	2,74128042	7,3588E-06
TMEM200A	129,774722	5,54467028	7,3588E-06
CCR7	2643,10572	2,81491304	7,4221E-06
KIR2DL1	377,766537	5,18185046	7,4221E-06
DUSP4	1450,15958	2,89570862	0,00001651
LTA	3108,29769	1,72288657	2,1253E-05
ITM2C	5256,17679	1,57221006	4,2724E-05
H2BC8	413,692085	-2,7678871	4,2724E-05
RIPK3	113,136687	-4,1245	4,2724E-05
CD82	452,459643	3,64525217	4,4758E-05
RASGEF1B	2634,48764	1,59744873	5,0095E-05
EPAS1	1129,94184	2,99668653	7,4428E-05
IKZF3	1813,06581	-1,3450101	8,1769E-05
TCEANC2	174,169466	-3,6381815	9,3006E-05
E2F2	208,363193	-3,7922519	0,00010067
IKZF2	505,167872	-2,4406656	0,00010067
CYTL1	276,562554	3,72969663	0,00010067

JAG1	746,137431	2,18326412	0,00012157
YPEL5	6148,8801	1,83574215	0,00012168
RGCC	384,13333	2,19599979	0,00012168
WDR3	272,525887	-2,3131554	0,00013701
KIF20A	560,961335	-4,2519971	0,00014288
SNRNP40	2658,42115	-1,1523362	0,00018837
SLAMF7	1053,26267	-2,252322	0,00019874
TSC22D1	601,477753	-3,3458403	0,00024087
CTC1	468,545718	-1,7023902	0,00033679
N4BP1	285,364395	2,25800969	0,00036013
THEM6	219,656732	-3,4099659	0,0003628
ZNF518B	191,074086	-3,261443	0,00052037
SIRPG	953,98196	-2,7325606	0,00057556
CD9	351,176444	4,25216552	0,00072844
DDX31	189,751674	2,45834394	0,000755
RASSF4	447,414768	2,98102576	0,00078717
METRNL	1418,8268	1,69010419	0,00082316
SH3TC1	96,2447839	-3,8174702	0,00083836
RNF130	364,376928	3,14416297	0,00108914
INTS6	1098,8311	1,26698675	0,00108914
CEP128	105,490925	-3,3972061	0,00108914
PRNP	1320,03293	1,34350406	0,00108914
MIR23AHG	1334,92099	1,36782291	0,00108914
MLX	769,469984	-1,1447829	0,00112764
NIBAN1	372,166095	1,93662096	0,00114148
GIMAP7	2986,3612	-1,431853	0,00141503
CD52	8974,1263	1,35832639	0,00148715
MSMO1	364,464322	-1,8690179	0,00155246
CAST	479,918342	2,0722165	0,00155246
GIMAP4	3468,21272	-1,1565038	0,00175945
SGK1	1490,76979	2,201372	0,00182347
EARS2	209,256727	-3,3722443	0,00182347
GNA15	315,844004	3,428828	0,00182347
IL10	93,8914481	-5,842076	0,00190438
MAPKAPK5	250,304684	2,02080611	0,00190438
UHRF2	311,706829	-1,6112519	0,0019864
CCDC18-AS1	698,987206	1,85509077	0,00206789
PLCB2	438,046477	-2,1952466	0,00219061
ACTRT3	91,7987225	-6,2312143	0,00257039
ATP2B1	1788,24329	1,20238505	0,0029875
CD44	13400,4781	1,12187744	0,00325416
LGALS1	4516,31434	2,00996322	0,00329528
IL1RN	262,958137	3,16576771	0,00357816
HLA-DRB1	149,874006	2,6856538	0,00357816
NCKIPSD	118,976609	-3,778849	0,00381578
NUDT16	145,290685	-3,2508719	0,00401251
SYNGR1	491,791439	1,81695781	0,00401251
ZNF45	214,939648	4,28212815	0,00408427
AIFM2	68,9606045	-4,6271653	0,00413915
SORL1	1274,64071	1,53221071	0,00413915
ZNF667-AS1	364,945895	2,04566131	0,00413915
VIM	935,985821	1,10924155	0,00416751
HDAC7	388,859688	-1,6435147	0,00416751
SKIL	4267,58717	1,55098469	0,00419473
LINC01871	723,907563	-1,3874009	0,00423677
PPP1R8	88,4644879	-4,1003735	0,00449512
RUBCN	170,065661	-2,4585976	0,00460812
NEIL3	164,361168	-3,5039636	0,00504009
CENPL	304,041503	-2,9573534	0,00516958
LDLRAD4	978,832911	2,44559847	0,00540114
DNAJC3	1710,03366	1,06660381	0,00547816
PLP2	501,28386	1,81154297	0,00592829
MYADM	1142,51937	1,72821378	0,00633276
GIMAP2	602,959431	-1,6002231	0,00648248

ELOA	1566,03178	1,05670243	0,00676193
MCEMP1	351,882662	3,32664023	0,00687565
THBS1	557,812345	-1,7610381	0,00738914
KIR3DL2	227,730153	3,85128196	0,00750273
GTSE1	368,178791	-3,112215	0,00770687
DENND10	216,890031	-1,9677488	0,00808931
ABCD4	826,105107	1,30255906	0,00831906
RNMT	707,356195	1,38915233	0,00861565
WDHD1	484,378954	-1,3910189	0,00945759
UBE2T	893,99532	-2,3856022	0,0095003
HSPB1	1874,52403	-1,9367133	0,00958051
GIGYF1	583,087279	1,24204136	0,00958051
TGDS	276,383863	-1,6430537	0,00958051
PFKM	103,429606	-3,0679187	0,00958051
ESD	879,289072	-1,0983672	0,00998106
SFXN4	326,221789	-2,6218935	0,01051875
RPAP1	213,099356	-2,6514579	0,01078998
DARS2	260,823907	-3,127235	0,0113143
GPR18	2506,45439	-1,0687711	0,01141127
SLC1A5	804,826126	2,00300459	0,01220685
ING1	501,894974	1,6077967	0,01228056
SLC20A1	2576,66531	-1,0910588	0,01241966
ZC3H14	829,141357	1,10358182	0,01241966
TCF3	192,987055	2,52317306	0,01241966
SLA2	1923,79092	1,09750157	0,01241966
ADAR	1136,84076	-1,136218	0,01254449
CAD	188,373713	-2,933279	0,01254449
KLF10	1575,5945	1,41584924	0,01254449
MAFF	3414,44239	1,41814183	0,01275991
MYO19	361,660912	-2,1475736	0,01290553
DFFB	86,4084483	-2,7433798	0,01305857
FTH1	7461,90046	1,06856433	0,01305857
ARAP2	941,167031	1,35932051	0,01316068
H2BC11	298,639094	-3,258137	0,01347966
LINC01353	171,298961	2,46270798	0,0137111
TPX2	1567,45982	-2,6326572	0,01379316
ODR4	249,082102	1,71941874	0,01384194
STARD9	135,043008	2,58877693	0,01446367
PSKH1	117,056471	-4,6387267	0,01469386
DXO	797,751968	1,67902034	0,01485014
KIF22	2876,24812	-1,3167212	0,01485014
ABLIM1	2054,88755	1,00569544	0,01536785
MAPKAPK3	1533,45107	1,32042479	0,01604958
NUFIP1	175,401148	2,33669792	0,01604958
SALL4	246,793089	2,84007719	0,01606242
VWA5A	144,331726	-3,5034913	0,01657071
TNFRSF11A	173,512412	2,82868948	0,0167097
GFM1	561,675916	1,23140504	0,01726078
WNT11	164,747057	2,709059	0,01726078
SCD	352,708973	-2,6473203	0,01732
TESC	630,655504	1,73404122	0,01755025
FAM111A-DT	351,510297	-1,9797224	0,01789873
EPM2AIP1	361,278964	1,51042963	0,01812386
GCC1	248,227767	2,61480818	0,01819982
ETF1	1633,75069	1,04342113	0,01848397
DNTTIP1	286,560901	2,24811446	0,01849761
CCNJL	117,400963	-3,5055976	0,01908044
FBXO21	773,131031	1,28767188	0,01908044
RAD54L	188,161998	-3,3920191	0,01920504
PIGC	825,259658	-1,8140826	0,01920504
H2BC9	590,297911	-1,5750509	0,01920504
R3HCC1L	808,566582	1,396791	0,01920504
SLC11A2	226,372209	-2,6620232	0,01920504
STX4	370,641433	1,44716985	0,01920504

DLG5	230,107107	-1,7262952	0,01927046
P2RY8	535,904224	2,05679028	0,01934022
INSR	116,736763	3,17071557	0,01953418
ATP9B	512,597024	1,48261635	0,02022233
RFK	236,941546	1,63314134	0,02042146
ARL4A	790,990519	1,07293874	0,02043322
ENTPD1	550,22045	-1,8911591	0,02063983
POP4	1662,32576	-1,2066832	0,02063983
PRRC2A	287,224525	-1,5440035	0,0206835
ST3GAL1	1626,47501	1,42385003	0,0206835
COG5	451,89837	1,02749456	0,02071659
RELL1	1156,60811	1,29081246	0,0219149
PTPN9	187,645339	-3,6333221	0,0219149
WWP1	278,266654	1,55101168	0,0225696
H2AW	360,366685	-1,7214665	0,02274144
ATAD2	651,549505	-1,7505429	0,02274144
MOAP1	1504,27146	1,01679349	0,02304781
ABHD4	830,368031	-1,7692697	0,02317365
MAP2K3	1982,36933	1,41134753	0,02317365
IL17C	136,358095	4,18662342	0,02317365
FXYD5	1605,66526	1,33276558	0,02317365
CAPG	1728,82739	3,27872035	0,02335099
TRMT2B	195,329325	-3,1234871	0,023423
FAM83D	196,53239	-2,4889737	0,023423
CHI3L2	170,350447	-2,5977547	0,02353032
TENT4A	277,017177	2,31587513	0,02353032
TULP3	159,099904	-3,3190344	0,02353032
RCBTB2	567,190949	-2,2350035	0,02353032
HAPLN3	165,30264	3,04561556	0,02353032
BTG3	811,292724	-1,0466624	0,02353032
ZBTB38	238,366561	-2,74017	0,02378708
ZNF346	355,450518	-1,5943714	0,02378708
LINC-PINT	664,784902	1,38643346	0,02378708
FANCG	500,937313	-2,7085343	0,02458405
KLRC2	764,554662	1,15434479	0,0250913
CD83	14487,9275	1,18092519	0,02565301
PTPRA	291,748481	-2,0564283	0,02571132
PDE12	335,407495	2,01787202	0,02604827
FOXK1	200,716543	2,4421052	0,02604827
FAM177A1	4305,96748	1,29395054	0,0274803
RAB3GAP1	176,497172	2,53762798	0,02823167
NOC3L	276,273457	-1,2961727	0,02823167
RANGAP1	690,957113	-2,1658748	0,02826182
H4C9	169,215998	-1,8119154	0,02920336
G6PC3	369,884026	-1,8358166	0,0302813
AGO2	1142,18131	1,16400572	0,03075758
CACNA2D4	264,848888	2,60419391	0,03082302
UCK1	223,167014	-2,7114684	0,03153582
PRDM2	918,548306	1,01117109	0,03195445
LINC00892	1321,39733	-1,4070764	0,03224633
IFT57	420,90913	1,08200662	0,03261234
TMPO	824,783273	-1,7073403	0,03261234
AREG	3478,76494	1,51335578	0,03281896
SIMC1	191,663751	3,2082831	0,03281896
KDM5B	331,020557	1,96333669	0,03427934
ASF1B	1744,54706	-1,4114792	0,03507397
GPR183	4107,5043	1,44330358	0,03530546
WIPI1	300,842316	1,84313432	0,03530546
BLVRB	263,064793	-1,63191	0,03631834
CTNNB1	6879,46331	1,03844963	0,03655879
MKI67	1226,01455	-1,8070028	0,03712178
HEATR1	292,901736	1,98514159	0,03737743
MRPL17	411,42117	-2,2925354	0,03796141
ECE1	367,966559	2,24191895	0,0379722

TNFRSF25	593,844816	1,32528691	0,03805019
TFIP11	599,728865	1,58371351	0,03805019
CCNB1	1288,3006	-2,387859	0,0382026
NIPSNAP3A	495,469816	-1,1423489	0,03862097
P2RX5	432,957102	-1,2421163	0,03871058
BUB1	865,374622	-1,5181031	0,03985718
JOSD1	676,188681	1,42268276	0,04033856
HSPH1	2076,97534	-1,6122823	0,04080931
NADK	438,503034	1,32640652	0,04105198
RORC	270,210758	-2,8423562	0,04105198
CCNA2	830,915903	-2,1920996	0,04105198
SAMSN1	2038,90801	1,39599256	0,04125577
SGPL1	1057,0542	-1,086411	0,04188039
GIMAP1	138,440347	-2,4257928	0,04195071
CDC45	944,72977	-1,8825875	0,04202569
NR4A3	1034,61282	1,76786112	0,04212151
CLPX	415,529514	-1,5532593	0,04212151
COQ9	699,437527	-1,0998102	0,04212151
H2AC6	1774,64141	-1,3528338	0,04257689
MXRA7	98,1950263	2,83049423	0,04257689
LEO1	814,002266	-1,8322716	0,04261039
ATP6V0E2	291,815886	1,50590062	0,04286451
WARS2	431,781593	1,24307451	0,04305671
PCID2	889,602381	1,1636543	0,04494643
GSTA4	169,085582	2,73753332	0,04557711
CNTROB	190,724325	-2,7052034	0,04557711
WDR91	204,424461	2,90513425	0,04559161
CHSY1	135,321993	-2,1520017	0,04577883
TUBG1	881,293664	-2,3994306	0,04602577
CDKN1A	909,452712	1,10017094	0,04642711
MOSPD1	222,773612	1,557497	0,04642711
KIF15	769,273575	-2,1625205	0,04719989
EHD4	427,106337	1,52092465	0,04719989
RRBP1	254,867179	1,38089419	0,04719989
GMPPB	749,372457	-1,8307045	0,04736297
ERO1B	181,591994	1,60170306	0,04792
LTB	3860,96118	1,11497207	0,04806327
SVIL	454,890288	1,05964823	0,04807917
FOXM1	157,505768	-2,4776484	0,04807917
FANCM	144,950496	-2,4876896	0,04807917
PPP1R21	123,060284	-2,449799	0,04834457
BCOR	654,331669	1,27357165	0,04850157
DUSP22	354,500068	1,86403997	0,04941532
TAF8	774,688864	1,33200472	0,04941532
HSP90AA1	32498,5108	-1,1781162	0,04980156
PDE6B	723,07572	-1,1538355	0,04992426
GRINA	305,975016	1,52675808	0,04992426

SKIN VS LUNG

Genes	baseMean	log2FoldChange	padj
DFFB	86,4084483	-4,62467	3,89E-06
ANGEL1	168,317653	-4,5957941	1,0464E-05
DUSP5	1273,78239	-1,662455	1,4103E-05
PFKM	103,429606	-4,3122866	0,00014784
RTL10	187,908859	3,30345003	0,00019614
CTC1	468,545718	-1,7975874	0,0003887
DLG5	230,107107	-2,4818655	0,00040429
IPP	144,180438	-3,1353307	0,0007949
ZNF518B	191,074086	-3,3305366	0,00099675
E2F2	208,363193	-3,5428858	0,00101874
E4F1	256,425702	-3,9407676	0,00101874
UHRF2	311,706829	-1,7826257	0,00111865
CD9	351,176444	4,36954782	0,00130643
RIPK3	113,136687	-3,5335189	0,00210079

CCAR2	352,160582	1,80968112	0,00305489
THEM6	219,656732	-3,1824996	0,00305489
TCEANC2	174,169466	-3,0840352	0,00465273
FGGY	180,927268	-3,9373768	0,00474764
PIK3R6	359,227803	4,69863058	0,00554167
ADPRS	615,504381	1,76332331	0,00706212
ADAR	1136,84076	-1,2935525	0,00764413
OTULIN	751,362827	-1,4237015	0,00865489
CHID1	1353,2118	1,20666989	0,00865489
CRY2	168,097636	-2,2042652	0,00865489
P2RX5	432,957102	-1,608726	0,00865489
CNOT10	776,524489	1,14041333	0,00959978
ANKRD49	608,280069	1,39202021	0,01346258
ITGA1	157,370327	4,72684685	0,01363498
NCOA5	208,840588	-3,0076448	0,01363498
HLA-DRB1	149,874006	2,58796001	0,01468612
PNKD	1482,46827	-1,0080351	0,01562918
PRDX4	943,093421	1,13920668	0,01581415
CRYZL1	836,580457	1,56179375	0,01719867
UBE2Q1	140,724377	2,46130858	0,01749041
MAML2	91,516854	3,18240908	0,01749041
RFLNB	174,218677	2,73747326	0,01993731
PLCB2	438,046477	-1,9369935	0,02265054
TASP1	458,749092	2,3460846	0,02265054
BRPF1	133,984297	2,74330779	0,02418616
HECA	382,652357	-1,4412243	0,0251058
IKZF2	505,167872	-1,7631813	0,02525581
PPP1R8	88,4644879	-3,7055933	0,02915102
DPP3	693,614246	-1,8922107	0,02915102
DCUN1D2	113,674465	3,97856225	0,02915102
DDX31	189,751674	1,90796941	0,03294486
TMEM200A	129,774722	3,33304419	0,03373276
PCED1B	688,211521	1,35013531	0,03625114
PSKH1	117,056471	-4,5553878	0,03625114
BTN3A3	261,294422	-2,2399201	0,03817
LMNA	2802,19063	1,88117559	0,03835377
SEC22C	406,607602	1,6158105	0,03925085
CLPX	415,529514	-1,7301713	0,03925085
MRPL43	592,25459	1,1189833	0,04395283
CEP128	105,490925	-2,6283807	0,04477233
NSDHL	296,747811	1,90825536	0,0454399
ELK3	346,931974	1,22042824	0,04741775
SGSH	539,144415	1,87449702	0,04741775
RANGAP1	690,957113	-2,2341251	0,04759515

CD16^{+} NK cells differentially expressed genes padj < 0.05 log2foldchange > 1 or < -1

INTESTINE VS LIVER

Genes	baseMean	log2FoldChange	padj
CAPG	173,4354853	3,904354837	9,40183E-08
CDCA3	432,3691091	-2,176505575	1,04461E-05
KIR3DL1	2823,343479	1,225590157	3,51916E-05
UBE2C	1557,423389	-1,453545819	0,001182815
CCNB1	542,0986453	-1,838870773	0,002178365
KIR2DL1	2210,534425	1,431707562	0,004850354
YES1	634,8062184	1,200951348	0,005200474
PLK1	668,649487	-2,352441957	0,00537335
H2BC11	157,9732983	-3,720784696	0,0062454
CCNB2	997,0825808	-1,250087527	0,0062454
YWHAH	278,1819562	-1,844314065	0,0062454
NOL6	263,4244093	-2,690109566	0,006435611
TASP1	366,2375168	-1,728230941	0,006435611
EGR1	1993,65636	1,716631227	0,009132637
TMBIM1	2020,179782	1,557750153	0,009574231
UBR1	395,6430245	1,495968153	0,009574231
SLC38A7	129,3394346	-4,801672702	0,010522253
MYRF	89,12814388	-4,661653677	0,013607371
TDRKH	324,3911765	-1,491551805	0,016783244
FLOT1	950,4836965	1,306538321	0,016783244
RASA3	1043,812589	1,18132552	0,017485851
KPNA2	2691,2163	-1,053130238	0,01807696
IFNG	11228,22235	-1,156168446	0,01916835
CREB3L4	164,8657464	-2,413788377	0,020267863
IMPDH1	507,1118045	1,674703401	0,020267863
LINC00892	373,6747578	-1,713659065	0,020267863
APBA2	470,6491693	2,400126437	0,020267863
GPR132	545,0428596	2,021160113	0,024485247
CDC20	1031,777886	-1,460547608	0,025932696
KIR2DL3	2187,931275	1,495919535	0,0300506
MELK	320,5271644	-1,686352373	0,032975903
AKR1C3	177,018259	1,930650854	0,032975903
DDHD1	456,2875513	1,1251502	0,037970691
CCNA2	482,0998804	-1,241321713	0,039112598
H2BC8	357,7779329	-1,462826149	0,039112598
TSPYL2	1839,374453	1,114971958	0,039112598
SYT11	259,3205182	2,198366315	0,040403296
PPM1L	147,85105	2,244847924	0,041198335
CXCL3	413,7356132	-2,235695493	0,042723847

LUNG VS INTESTINE

Genes	baseMean	log2FoldChange	padj
CAPG	173,4354853	-3,641059019	2,4106E-06
TASP1	366,2375168	1,835747252	0,00761911
CDCA3	432,3691091	1,677758381	0,00826226
PAN2	246,0757242	-2,102331258	0,00867295
KIR2DS4	1816,412682	1,375018391	0,01425884
CSF2	640,7066702	2,809198638	0,0225184
BOP1	400,4863817	1,348205308	0,03518636

LUNG VS LIVER

Genes	baseMean	log2FoldChange	padj
KIR3DL1	2823,34348	1,60694781	5,7659E-10
KIR2DL1	2210,53443	1,89708218	5,4029E-06
CD27	424,484561	-2,0801559	1,2844E-05
KIR2DS4	1816,41268	1,84819499	1,2844E-05
SIRPG	604,1359	-3,1575173	0,00031329
GIMAP6	585,373268	-2,6566653	0,00033959
GSTP1	10782,7441	-1,0332951	0,00110352
LINC00892	373,674758	-2,0566614	0,00155459
H2BC8	357,777933	-1,8374096	0,0018913

METRNL	1395,71784	1,4809298	0,00335346
KIR3DX1	216,660591	2,56086235	0,00335346
GZMK	1676,00808	-1,4722492	0,0041336
KIR2DP1	456,695341	2,08927101	0,0052614
KIR2DL3	2187,93128	1,70717189	0,0052614
PPM1L	147,85105	2,53896173	0,00967645
APBA2	470,649169	2,53073262	0,00967645
GZMB	44455,3692	1,09090811	0,01154129
NEB	750,914555	1,62609724	0,01336974
CSF1	180,657393	-3,1288818	0,0141395
CXCR6	1120,96731	-1,9163297	0,0141395
H2BC11	157,973298	-3,3570506	0,01496364
YES1	634,806218	1,06109644	0,0153399
SLC12A6	131,25334	2,42931342	0,01652487
PDE6G	788,625204	1,13976943	0,02075619
EGR1	1993,65636	1,55425855	0,02104022
ARRDC2	592,047483	1,52754574	0,02104022
H3C10	550,06846	-2,1538133	0,02171711
GPR132	545,04286	1,97708084	0,02568858
GNPTG	621,691245	1,28598733	0,03144873
YWHAH	278,181956	-1,5599941	0,03144873
PRKAR1B-AS1	185,738948	2,37593312	0,03383998
DNAJB4	565,296656	-1,7085263	0,03892474
MAML1	296,801366	-1,4581157	0,03892474
TAZ	683,727421	1,51105814	0,03892474
UBR1	395,643025	1,29154117	0,03965844
AGO2	908,145232	1,28985231	0,04056332
LPAR6	800,412365	-1,2062584	0,04086522
TADA1	572,069439	1,2303699	0,04376239
SYT11	259,320518	2,14470452	0,04440542
SMARCAD1	230,418538	-1,2293514	0,04440542
YKT6	375,350497	1,84611943	0,04440542
TRMU	478,483011	-1,703127	0,04440542
TIGIT	1420,45304	-1,2353697	0,04452989
PHF21A	579,581129	1,06020699	0,04530857
RRBP1	254,268109	1,55877418	0,04541359

SKIN VS INTESTINE

Genes	baseMean	log2FoldChange	padj
CREB3L4	164,865746	3,60597679	1,2024E-05
CDC20	1031,77789	-1,9973574	0,00046063
CAPG	173,435485	-3,0298787	0,00046063
KIFC1	152,832497	-3,1342602	0,00191519
GPA33	157,618378	4,62337192	0,00263872
C1orf112	177,372988	-2,5585224	0,00266061
HSD17B7	447,255051	-1,4660911	0,00266061
KIR2DS4	1816,41268	1,4417019	0,00423446
LIMK2	419,359333	2,29623329	0,00473529
CHID1	1027,29812	-1,1761432	0,00765771
CDCA7	966,403573	-1,1653041	0,01060179
BOP1	400,486382	1,40912992	0,01060179
NOL6	263,424409	2,59304849	0,01093554
KNSTRN	266,38749	-2,1076961	0,01635673
PCK2	177,196316	-2,1612994	0,02110491
UBE2C	1557,42339	-1,1940402	0,02151535
CSF1	180,657393	-3,4953101	0,02177544
ACOT7	1041,13875	-1,1973804	0,02234001
FRMD4B	212,232081	-2,7900304	0,02234001
IRAK1	232,595612	-1,6336272	0,02234001
DIP2B	319,29826	-1,6944973	0,03225468
USF3	377,787184	1,49374281	0,03307748
ADCK5	109,358202	-3,5844328	0,03307748
THBS1	403,775114	-2,7597097	0,03307748
SIRPG	604,1359	-2,372846	0,03307748

GRAMD1A	256,723428	-2,2267929	0,03671359
LSS	212,564584	2,99335246	0,04224287
ENPP4	305,1983	-1,8093651	0,04429771
INTS3	339,079323	1,32347927	0,04568201
IL1RL1	413,227484	-2,0466987	0,04568201
PML	415,353588	-1,7496153	0,04952781
HJURP	455,666258	-1,5724738	0,04992815
CYSTM1	327,451531	-2,0820481	0,04992815
ERCC6L2	258,448459	-1,894165	0,04992815

SKIN VS LIVER

Genes	baseMean	log2FoldChange	padj
KIR3DL1	2823,34348	2,09254008	1,3762E-18
CDC20	1031,77789	-3,457905	5,9862E-16
UBE2C	1557,42339	-2,647586	1,3104E-15
CCNB2	997,082581	-2,1161042	1,8553E-10
SIRPG	604,1359	-4,3936419	2,4349E-09
KIR2DL1	2210,53443	2,16758757	4,2152E-09
CSF1	180,657393	-5,8654164	1,4922E-08
CDCA7	966,403573	-1,8402035	1,6149E-08
H2BC11	157,973298	-6,3440884	1,6299E-08
KNSTRN	266,38749	-3,1616758	4,3634E-07
KIR2DS4	1816,41268	1,9148785	1,0103E-06
CXCL3	413,735613	-3,8019539	1,3383E-06
KIR2DL3	2187,93128	2,3175628	1,4881E-06
CD27	424,484561	-2,1180157	2,3595E-06
C1orf112	177,372988	-3,1368904	2,8164E-06
H2BC8	357,777933	-2,3164037	3,0045E-06
MELK	320,527164	-2,6278711	3,0711E-06
ID3	356,398649	-3,747046	1,7018E-05
STARD3NL	2511,04089	-1,134778	3,0608E-05
KIFC1	152,832497	-3,4384226	3,0651E-05
CCNB1	542,098645	-2,0795313	3,2937E-05
RIPK2	1477,1248	-1,1592454	5,2407E-05
CXCR6	1120,96731	-2,4665747	7,0599E-05
HSD17B7	447,255051	-1,6016901	7,1098E-05
UBR1	395,643025	1,78301563	0,00011659
KIR2DP1	456,695341	2,42403757	0,00011659
RHBDF2	403,940335	2,03674743	0,00018015
SMARCAD1	230,418538	-1,720403	0,00018372
H3C10	550,06846	-2,7846312	0,00018584
IBA57	205,587022	3,63833745	0,00021236
CDCA3	432,369109	-1,7963515	0,00022602
LINC00892	373,674758	-2,1291132	0,00024495
HSH2D	2986,0956	1,20794225	0,00026762
ACOT7	1041,13875	-1,4666388	0,00027734
PLIN2	9138,85229	-1,1356562	0,00028154
KPNA2	2691,2163	-1,2592424	0,00029103
UTP25	267,089706	2,38192323	0,00029781
IL12RB1	602,640354	1,90757563	0,00030554
QRICH1	2177,43676	1,35496103	0,00031318
INTS13	413,967752	-1,7569593	0,00032453
CCNA2	482,09988	-1,5549159	0,00060987
TRDJ2	241,313739	-1,8439719	0,00060987
SYNGR1	517,899983	1,48010585	0,00063579
ACTR1B	420,696528	-1,8571288	0,00064866
METRNL	1395,71784	1,51688864	0,00064866
ZWINT	1994,66117	-1,002584	0,00066741
GZMB	44455,3692	1,2102943	0,00073705
TMBIM1	2020,17978	1,66922106	0,00073924
ZNF683	495,20095	2,14004921	0,00105188
NLRP1	1650,88923	1,10499038	0,00113289
PAICS	690,185522	-1,677844	0,00122835
GART	1064,2178	-1,3395852	0,00156514

CCL25	55,9031181	-6,9156524	0,00168457
ACSF2	97,6670523	-4,9457156	0,00186423
TNFRSF25	486,256962	1,92967871	0,00210927
IFNG	11228,2224	-1,2384729	0,00210927
ATP6V1C1	592,846542	-1,0996334	0,00213399
RNF166	1175,17268	1,78578741	0,00213399
CRELD2	1090,26264	1,10841744	0,00213399
VPS51	672,39431	1,77727951	0,00214728
KDM3A	571,479322	1,71233554	0,00256991
C1orf131	362,499302	-1,3796364	0,00256991
DCAF12	383,137853	-2,2923307	0,00256991
B9D2	215,835391	1,76957195	0,0026317
HJURP	455,666258	-1,8514764	0,00273302
NEK8	108,667364	4,00189511	0,00273302
SERPINH1	197,078271	-5,2453122	0,00274248
ZBED4	409,168479	2,38990136	0,0028294
AKR1C3	177,018259	2,13259886	0,00293584
DNAJB4	565,296656	-1,9431574	0,00303969
SLC30A5	219,294832	2,03188152	0,00305806
GZMK	1676,00808	-1,3756565	0,0032987
PTTG1	1218,56845	-1,2573464	0,00346027
ADCY7	565,505037	2,0933751	0,00392574
PLK1	668,649487	-2,1362524	0,00392574
PSRC1	118,534501	-4,4635068	0,00397451
CENPW	428,558551	-1,1605217	0,00397451
P2RY8	983,233998	1,88179635	0,00397451
RASSF4	1238,01455	1,40338581	0,00397451
SMC2	620,800411	-1,2049173	0,00431938
CDK4	788,322313	-1,2820074	0,00431938
AGO4	176,527511	2,38370873	0,00443802
TLE5	2015,66502	1,18386969	0,00485591
THBS1	403,775114	-2,8942825	0,0052698
HMMR	163,2216	-2,8767168	0,00590324
MON1B	751,902921	1,79232994	0,00596442
RRN3P1	412,035921	1,24378594	0,00614347
SLC5A3	330,1504	-2,3859965	0,00614347
MVP	575,004077	2,29939017	0,00619372
CKS1B	953,973804	-1,0751573	0,00660749
CDKN2B	88,6633804	3,7043299	0,00660749
CHID1	1027,29812	-1,0490585	0,00716868
RRBP1	254,268109	1,68172611	0,00754184
PRKAR1B-AS1	185,738948	2,45375302	0,00783574
PHF8	310,190869	2,03426744	0,00783574
GPN3	423,075827	-1,5633973	0,00783574
CYBA	1601,34694	1,24578774	0,00783574
GCLM	113,656537	2,27411983	0,00800345
DPY30	812,423041	-1,087536	0,00800345
SLC25A17	223,305135	-2,0726675	0,00800345
RGS9	470,393979	1,7732284	0,00811675
SLC43A1	336,936878	1,65128082	0,00893505
HSPA7	389,104056	-2,9397065	0,00895509
FGFBP2	2516,89339	1,5447402	0,00921928
PTGES3	1845,81224	-1,0142232	0,00921928
CALCOCO1	1264,56398	1,11126868	0,00937112
CIT	172,088347	-2,4533316	0,01012276
COLGALT1	1221,305	-1,276575	0,01012276
CXorf38	338,873416	1,77041435	0,01016734
S1PR1	1036,13848	1,56074978	0,01021884
LAIR2	278,133669	1,81665712	0,0103877
CD28	267,30928	-2,5467971	0,01046916
KIR2DL4	1786,07855	1,27095531	0,01046916
MIR181A1HG	720,351746	-1,1278288	0,01060384
NEB	750,914555	1,49114641	0,01060384
ZFC3H1	1146,07821	1,12591443	0,01060384

ZNF766	353,95506	2,08623664	0,01060384
ATF6B	642,61256	1,32025391	0,01086856
IKZF5	1011,34734	1,00017005	0,01098281
SPC25	201,287961	-1,9158194	0,01138626
CKS2	1850,55949	-1,2701855	0,01138626
USP37	163,168149	-2,3484529	0,01273603
HS3ST3B1	207,523909	2,44121457	0,01273603
BOLA1	143,097492	-3,0548686	0,01313983
FBXO22	444,180252	-1,4998234	0,01313983
PNP	447,833588	-1,5622471	0,01313983
COA3	1679,32992	-1,1420934	0,01313983
YJU2	755,284828	1,11860436	0,01313983
GPR132	545,04286	1,88692274	0,01343195
H2BC21	1105,10344	-1,1190813	0,01345973
GCC1	232,39077	2,54340829	0,01362037
ARID5A	2430,44766	1,0223822	0,01408663
LDLRAP1	275,828532	2,67648886	0,01410394
KNL1	255,110187	-1,779467	0,0143098
ZDHHC16	296,480332	-2,5798146	0,0152133
EMG1	965,081624	-1,0258572	0,0152133
SPON2	2642,4674	1,03278475	0,01529418
PSMB5	1077,66138	-1,1299498	0,01542629
TMEM99	172,742218	-1,7736231	0,01542629
SYTL1	1856,88516	1,04322486	0,01556272
KDM2B	736,59686	-1,0726413	0,01569439
AGBL5	191,788631	-2,692896	0,01604486
PEX11B	402,378063	-1,3561347	0,01619551
ATXN7L1	269,94421	1,77625964	0,01629528
TROAP	435,593599	-2,2797026	0,0164324
PTGER2	430,632841	2,95109587	0,0164324
EMC10	376,42639	1,40092369	0,01664517
CCDC127	390,022601	-1,5585966	0,01677725
IRAK1	232,595612	-1,4703408	0,01677725
GTF3C1	1099,79077	1,28622925	0,01764612
GTSE1	177,140875	-1,6236507	0,01764612
RNH1	2051,37992	1,04490502	0,01818125
ERCC6L2	258,44846	-1,8636138	0,01829285
IL21R	501,957145	1,59920901	0,01847762
GRAMD1A	256,723428	-2,094172	0,01847762
MGAT5	132,052404	-2,257214	0,01853913
CACYBP	4229,10361	-1,2791029	0,01875077
PHF21A	579,581129	1,03431663	0,01875077
SESN1	3039,02686	-1,2825409	0,01912007
PPIL1	256,916518	-1,5516743	0,01935878
IDS	581,181869	1,2391677	0,01935878
P4HTM	497,012954	1,41591322	0,0198371
IL1RL1	413,227484	-1,9670404	0,01984351
CAST	517,790647	1,49352944	0,01987839
AP3B1	537,136868	1,11340649	0,01988715
ARRDC2	592,047483	1,36346623	0,01988715
PRSS30P	276,676491	5,0744684	0,02006778
HSPB1	2346,04356	-1,4003859	0,02011945
CCDC90B	664,671031	-1,1409365	0,02026925
INPP5K	2700,05499	1,01179212	0,02119094
ZNF383	170,692589	1,77465515	0,02119094
PDE6G	788,625204	1,00321875	0,02180046
SLC38A7	129,339435	-3,8648006	0,02184563
FANCI	639,266761	-1,4808833	0,02321605
ZFAND2B	1720,77088	1,16849952	0,02358336
HEXIM1	299,629756	1,29858935	0,02358336
CAPN2	1369,56685	1,02229919	0,02420438
OXLD1	355,415213	1,30141434	0,02520494
ARFIP1	274,577054	-1,176496	0,02573511
CCDC6	271,258675	1,3068027	0,02650672

NUP43	312,465287	-1,3389306	0,02674115
UBQLN2	657,739661	1,17346582	0,02674115
FAM76B	276,921196	1,46816845	0,02674115
NAAA	554,80564	1,42427262	0,02733311
PNPLA8	1494,14387	1,01784317	0,02740386
TIMP1	433,298898	1,53255861	0,02741257
CHI3L2	322,265571	-1,6992398	0,02751292
PGM1	671,136905	1,27663684	0,02751292
CTDSP1	430,083198	1,38193234	0,02751292
ATXN2	235,103895	-2,1433148	0,02751292
ZNF845	259,575712	2,59335028	0,02751292
SNX10	433,439353	-1,0968279	0,02875931
AXIN1	519,207115	1,06552194	0,02875931
TNKS2	355,8153	1,26744833	0,02958131
YKT6	375,350497	1,68795795	0,02974747
TSC22D2	474,738786	1,27746576	0,03003196
TPX2	947,691271	-1,2510623	0,03003196
TMED5	304,566344	-1,0458776	0,03018206
SLC4A10	1018,60769	-1,4071158	0,03122522
DYNC2I2	789,063026	-1,6235445	0,03130913
ISCA2	318,014003	-1,4144067	0,03143097
RTN4	839,381006	1,01429663	0,0315082
POLR3H	212,967592	-1,6492232	0,03238356
STIP1	2454,45452	-1,1187884	0,03384737
CISD1	284,582934	-1,1628664	0,03442344
SDF4	867,497901	1,04073239	0,03464519
CENPM	1343,45704	-1,401107	0,03531696
ZNF827	499,879813	1,02635549	0,0354204
SPTSSB	271,065614	-1,7726926	0,03560571
NCAPD2	988,533877	-1,2917044	0,03562076
NPRL3	380,65472	1,60428389	0,03594858
RAD51	437,698692	-1,3133284	0,03597681
RUVBL2	2021,00501	-1,1143953	0,03651527
UBE2T	434,850728	-1,2373125	0,03679085
SLC12A7	454,731	2,01238876	0,03680304
ATG2A	257,115889	1,87588527	0,03871256
KIF15	433,853954	-1,5542161	0,03957544
WNT11	433,773312	1,87910301	0,03957544
KIR3DL2	1077,81724	2,64944273	0,03957544
NLRP2	332,772946	-1,6638895	0,0399473
AGO2	908,145232	1,11649174	0,0401226
VPS26B	445,646163	1,38778592	0,04024352
ZNF75A	338,626253	1,42617613	0,04063833
SEC24D	256,518858	1,50086448	0,0407914
AHNAK	1083,05413	1,14608368	0,0407914
ATP2B1	1639,29145	1,17754874	0,0407914
HMGB3	320,784097	-1,2179708	0,04083634
TUBA1C	237,316987	-1,5678918	0,04083634
FAM193B	203,100648	-2,004589	0,04105743
PGRMC1	235,542322	-1,8002504	0,04118633
MRPS14	829,869603	-1,0008039	0,04197374
ABCB8	343,143746	1,63662213	0,04197374
SERPINB9	1685,59435	1,32412215	0,04267004
FAM50B	98,5711436	-3,0975748	0,04317926
BRPF1	222,3545	2,10751368	0,04331113
CHEK2	356,135537	-2,1458733	0,04331113
FANCL	225,770756	-1,8113646	0,04345123
RGS1	27231,2219	-1,0895576	0,04356911
IMMP1L	232,877142	-1,0946219	0,04356911
NFATC2	619,937687	1,05886796	0,04356911
PRR3	164,010402	-2,531464	0,0446317
TTC7A	182,073296	-2,1707504	0,04498408
INO80D	727,482173	1,37827107	0,04501681
ARSA	362,773584	1,83145212	0,04516181

RGS14	783,168443	-1,1836639	0,04640029
DIP2B	319,29826	-1,369488	0,04690061
TADA1	572,069439	1,04772846	0,04694921
NEK2	170,905153	-2,6735325	0,04695383
PPP4R1	363,895873	1,73252068	0,04699582
SNHG12	377,771823	-1,2645659	0,04735977
GPAA1	1369,16538	1,02690288	0,04737738
NUP37	389,112626	-1,2045099	0,04737738
TANGO2	639,428313	-1,2819916	0,04737738
ATG16L1	795,410508	1,11940919	0,04805032
VPS18	233,756417	1,56880717	0,04805032
RNF38	440,498488	1,23757185	0,04843213
MANBAL	396,763602	1,73125999	0,04860184
PIK3R4	305,514521	1,61411194	0,04875971
SLC22A18	235,979095	2,02704312	0,04969442
NLE1	219,961371	-2,879364	0,04974186

SKIN VS LUNG

Genes	baseMean	log2FoldChange	padj
CDC20	1031,77789	-2,8220592	7,1759E-10
UBE2C	1557,42339	-2,0055356	4,5572E-08
ZBTB39	73,3894067	-4,6633929	0,00026086
CCNB2	997,082581	-1,4800496	0,00044161
DIP2B	319,29826	-2,2683473	0,00048029
CDCA7	966,403573	-1,2974516	0,0024461
KIFC1	152,832497	-3,0366741	0,0024461
PAN2	246,075724	2,13512101	0,0042587
USP37	163,168149	-2,9043462	0,00449802
GTSE1	177,140875	-2,0474598	0,0060475
ACOT7	1041,13875	-1,3402467	0,00666501
PDPR	287,353765	1,71480607	0,00753879
MAN1B1	680,454972	2,38017762	0,00786166
LIPA	636,029193	1,20933106	0,00949603
WDSUB1	421,695901	1,34679178	0,0141947
CROCCP2	343,683218	1,96978886	0,0143302
GPA33	157,618378	3,8692282	0,01503725
HCCS	420,642309	-2,1411694	0,01503725
IRAK1	232,595612	-1,6803898	0,01503725
MELK	320,527164	-1,8025014	0,01967625
KNSTRN	266,38749	-2,0216446	0,02094865
ARL5B	1216,52252	-1,3861536	0,02246035
B9D2	215,835391	1,6383572	0,02273539
MED12	448,376738	-2,1901075	0,02540373
RC3H2	307,248772	-1,6089327	0,02752339
ZBTB40	213,725336	-2,4292797	0,0313421
TSC2	263,570546	-3,0462874	0,0313421
SIGLEC17P	342,975959	2,11867978	0,03363947
HMMR	163,2216	-2,7112496	0,03437942
URB1	65,5446232	-4,206318	0,03594169
KDM2B	736,59686	-1,1032188	0,03649641
CREB3L4	164,865746	2,13249123	0,0420722
ZSCAN26	361,390422	1,77001863	0,0420722
CSPP1	161,236	-1,6669989	0,0420722
CIAO3	198,09076	1,99878333	0,0420722
KNL1	255,110187	-1,7742921	0,0420722
ZBTB20	312,902004	1,37155613	0,04317956
TROAP	435,593599	-2,28285	0,04317956
ZNF576	124,260157	2,46948221	0,04317956
CDCA3	432,369109	-1,2976043	0,04485065
SCARB2	114,366943	3,24601161	0,04954054
ASH2L	669,426637	1,09969634	0,04954054

CD304⁺ ILC3s differentially expressed genes padj < 0.05 log2foldchange > 1 or < -1

INTESTINE VS LIVER

Genes	baseMean	log2FoldChange	padj
CYP26A1	1340,134827	4,85698102	7,90838E-27
NCR2	343,5739105	3,88569309	1,67927E-06
ALOX5AP	4612,008295	1,96504696	1,67927E-06
PLAT	114,6223706	5,35788757	8,4819E-06
NCAPG2	1968,111998	-1,699468629	3,65881E-05
DHRS3	5051,062567	1,661196283	4,82453E-05
ING4	442,6976429	-2,477853946	4,82453E-05
EMP2	96,83732178	7,305036849	0,000269343
SRPX	203,2987411	3,85075357	0,000322114
GSTP1	989,457287	1,939440062	0,000830854
ADTRP	413,3062109	2,497046351	0,000898482
SERPINA11	1349,69143	-1,569327995	0,000898482
LDLRAD4	629,4004044	2,247911214	0,001125028
SIRPG	220,2624206	4,037100882	0,001350916
CEACAM1	507,516072	2,110963581	0,001447103
PIEZO1	791,3748079	-1,695074377	0,004082108
APOL1	182,0704184	3,866503644	0,004573363
TSPYL2	2202,562649	1,479777924	0,007561836
BANK1	612,1795204	-1,595676794	0,010677831
PLK2	639,5590354	1,786531893	0,013330175
PIAS3	250,8438697	-2,277915439	0,016277559
BIN1	662,7968834	-1,742570841	0,017731123
CD38	576,214292	2,130962785	0,017731123
ZNF766	162,8613838	-3,188740803	0,018935968
FAM3A	225,9848356	-2,452857399	0,020052037
ADAM19	1186,821805	1,465026614	0,022011308
HPCAL1	872,9741731	1,494406755	0,02310528
CHST9	421,4823325	-2,049933129	0,02310528
THAP7	237,7769643	-3,17369692	0,02310528
DUSP4	222,2987965	2,393004568	0,02411495
REEP4	383,6940487	-1,739113195	0,02411495
SH3GL2	103,8051274	-4,751116873	0,02411495
SAFB	891,7309639	-1,305310945	0,02411495
PEG10	796,2409867	-1,557259172	0,027165921
ARMCX5	148,5170884	-3,002832071	0,027471337
TMEM38A	175,7411776	-3,369009754	0,027471337
G0S2	216,0890514	-3,183837829	0,028550169
CA6	520,2669815	-1,810256061	0,028550169
DNMBP	147,3768646	3,661568511	0,028550169
PI16	213,8386304	-3,501951704	0,029541989
INSIG1	1342,337206	1,295808073	0,031490284
PHGDH	315,8477363	3,207015791	0,03312792
GNG13	214,3453208	2,124100858	0,03449506
RASGEF1B	596,5045853	1,502613754	0,036278926
REV3L	446,6447282	-1,574935676	0,036433218
SMC1A	1286,637565	1,174586815	0,036433218
SEC13	2303,35593	1,116374301	0,036581919
TSPO	3131,081212	-1,173580693	0,036581919
BLVRA	1304,474185	1,071436514	0,036964778
DHCR7	1514,12027	1,068930409	0,036964778
GPATCH2L	359,169206	1,989785438	0,036964778
KIF16B	160,1858272	-2,52468049	0,038650971
CDCA4	298,5311315	-2,308050904	0,038962142
KRT2	166,4562344	-2,920485837	0,040650983
ATP13A2	119,404169	-3,945087999	0,042584921
CFH	2524,675025	-1,008068061	0,042642053
ABCB1	597,6094516	1,845469021	0,042642053
GFPT1	199,1780648	2,651368696	0,043179812
NEIL3	154,6195974	-2,671828934	0,043179812
CTSL	285,3567118	2,897249662	0,044059461

EXTL2	135,5655559	-2,535122495	0,04424256
ZYX	256,9960842	2,529009501	0,045043466

LUNG VS INTESTINE

**Genes**	**baseMean**	**log2FoldChange**	**padj**
CA6	520,266982	3,27359958	1,3179E-07
IL2RA	1356,35765	2,49981869	1,5676E-07
SMOX	355,157993	4,32918362	3,1042E-07
ENPP2	176,13227	4,78393152	1,2304E-06
ING4	442,697643	2,65089436	5,9114E-06
SERPINF1	256,639875	-3,0945085	0,00113817
FGR	4908,59248	-1,6495945	0,00771639
CFH	2524,67503	1,23648167	0,01254108
TNFRSF1B	683,449102	2,27533883	0,02123248
IMPDH1	211,416262	3,44252525	0,0254328
PPP2R5D	864,631743	-1,3942245	0,02603836
PFKP	340,366519	2,15975203	0,02876799
DHX16	417,29254	-2,4082643	0,02988894
CPXM1	312,707507	-2,0509834	0,03220098
SLC25A20	294,01542	-1,9472187	0,03630904
COG1	350,322106	-2,1220283	0,03630904
TGM2	1412,21676	-1,2965529	0,03658926
SH2D3C	209,543553	-2,8330173	0,03848418
EXTL2	135,565556	2,72989996	0,04220935
PRMT6	144,410325	-2,8861092	0,04220935
SOCS2	544,510627	1,50105548	0,04220935

LUNG VS LIVER

**Genes**	**baseMean**	**log2FoldChange**	**padj**
CYP26A1	1340,13483	5,47001474	1,0998E-34
IL2RA	1356,35765	3,34206789	4,4565E-15
SRPX	203,298741	5,77482209	2,7232E-11
ENPP2	176,13227	6,41727561	2,7232E-11
ALOX5AP	4612,00829	2,33576685	2,8357E-10
DHRS3	5051,06257	1,96704641	9,8598E-08
SMOX	355,157993	4,19036992	2,281E-07
SERPINF1	256,639875	-3,9817089	2,8599E-07
NCAPG2	1968,112	-1,8366154	1,1672E-06
FGR	4908,59248	-2,2254521	1,6178E-06
PLAT	114,622371	5,26749639	5,2277E-06
NCR2	343,573911	3,59278487	6,9719E-06
PTPN6	2165,3576	-2,4644291	7,5193E-06
WDPCP	243,040003	-3,1690608	8,2423E-06
PLK2	639,559035	2,39474093	2,2252E-05
EMP3	3326,75679	-1,6759539	2,4172E-05
SAMSN1	1356,93756	2,60484414	4,118E-05
ADAM19	1186,8218	2,00513208	4,5092E-05
COG1	350,322106	-2,8996684	5,9924E-05
TTN	3698,18809	-2,3256839	6,5109E-05
DEDD2	479,613427	-2,3245332	9,8896E-05
CHAD	435,542027	-2,8260955	0,000116
XCL1	3765,90302	2,15886233	0,00020345
PRMT6	144,410325	-3,737617	0,00030947
BIN1	662,796883	-2,1375517	0,00034216
PGGHG	1082,22945	-1,8011054	0,00040422
ZNF331	6695,53309	1,42472877	0,00040422
RIPOR2	943,053326	-2,2509334	0,00047724
LRRN3	311,563175	3,60825179	0,00051672
SHISA2	1915,73981	2,10402301	0,00053214
DDX23	623,626542	-2,2832208	0,00053911
ADTRP	413,306211	2,42440465	0,00065736
ABCB8	268,616873	-3,4090775	0,00076647
CTSL	285,356712	3,86035237	0,00076647
LDLRAD4	629,400404	2,1820287	0,00083762

HPN	3764,5022	-2,1540584	0,00083762
LDHA	14568,6358	1,35013303	0,00095497
IRF7	2180,41811	1,64088304	0,00095497
HPCAL1	872,974173	1,76990896	0,00101394
LYST	843,216661	1,59890493	0,00128985
LZTFL1	1554,22445	-1,5392431	0,00129152
TRAF4	523,126249	2,04275271	0,00129152
AMPD2	758,21677	1,59569194	0,00161556
KRT2	166,456234	-3,7537626	0,00161556
PGK1	11023,776	1,00355924	0,00165069
P4HA1	1237,68259	1,5872246	0,00177547
DHX38	452,598843	-2,4046544	0,00213866
SCN1B	1065,47146	-1,9768201	0,00213866
TNFRSF1B	683,449102	2,38327081	0,00226784
SRSF7	9309,1686	1,00248816	0,00226784
ZNF488	417,074949	-2,5282149	0,00226784
CDKN1A	922,559262	1,93251036	0,00239565
INSIG1	1342,33721	1,51503204	0,00268119
TBXAS1	262,88434	2,50063012	0,00270513
SELPLG	8713,08991	-1,1960849	0,00287375
PEG10	796,240987	-1,7651571	0,00307194
DHCR7	1514,12027	1,26627481	0,00314287
CASP3	8464,40089	1,21987825	0,00331272
VAMP5	2264,13392	1,15389305	0,0038016
KHDRBS2	268,847796	-2,3343103	0,00402023
ACAA2	3178,81513	1,08740114	0,00402513
TXNIP	4033,70901	-1,6570336	0,00482443
TSPYL2	2202,56265	1,43835373	0,00505325
ATP1B1	1090,48587	1,2850101	0,0053114
NCF4	332,229874	2,85651183	0,0053114
PIEZO1	791,374808	-1,561471	0,00565254
SLC39A8	434,874852	2,54465928	0,00575947
GTF3C5	521,070175	-1,8996292	0,00596257
CREM	8209,7768	1,51196809	0,0064386
ZIK1	203,949706	2,8299919	0,00675123
CDK16	610,015914	2,32345256	0,00710395
SKIL	1045,04245	1,47619291	0,00718608
BNIP3L	1921,38256	1,10117324	0,00718608
ARMCX5	148,517088	-3,201344	0,00718608
PPP1R9A	1657,51028	-1,314096	0,0075661
EIF5A2	176,844373	2,7897777	0,00772804
DDIT4	3089,53454	-1,1498925	0,00813034
COTL1	3241,65201	1,04676864	0,00831306
CTCF	143,432444	-2,8526672	0,00844274
IGFBP7	551,885359	-1,6893509	0,00872013
C3orf52	391,243546	2,17906144	0,00885912
PECAM1	3521,81345	-1,1577671	0,00901415
CTHRC1	294,293072	-2,0470152	0,00903467
PFKP	340,366519	2,11885098	0,00903467
CPXM1	312,707507	-2,0457373	0,00903467
PI16	213,83863	-3,6575121	0,01001622
MYO1F	2979,60989	-1,334577	0,01083911
CNEP1R1	257,344787	-2,2370562	0,01111844
RORA	1392,14296	1,33371452	0,01213874
CCDC26	139,173635	-4,0114928	0,01224596
C19orf48	2192,61259	1,52466371	0,01224596
NKIRAS1	913,634414	1,38771116	0,0125181
RNF146	236,743403	-3,1131213	0,01258744
TXN	2467,53495	1,03524065	0,01313353
UAP1	1484,73242	1,44418351	0,01559816
HPRT1	986,171734	1,23741422	0,0165325
GTF2F1	415,438582	-1,8324387	0,0165325
SAFB	891,730964	-1,2599515	0,0165325
TACC3	587,92124	-1,6560986	0,01749618

CLIC3	1111,59456	-1,1537383	0,01754915
TSPAN4	1011,87917	-1,4563333	0,01754915
DOK2	580,847742	-1,7618632	0,01920805
PITPNA	119,538836	2,88573135	0,01928636
SEPTIN11	827,830889	1,29540472	0,02014658
PPP2R5D	864,631743	-1,2583008	0,02046909
PFKFB3	1626,95078	1,08807749	0,02350742
ICOS	3133,82231	1,25164976	0,02438916
SYTL1	791,670225	-1,3102098	0,02479865
LMAN2L	1157,40048	-1,4947759	0,02541348
TRGV9	1511,00496	-1,0452895	0,02559099
PLSCR1	965,102827	1,14343794	0,02608169
GPX1	1102,29929	1,34407773	0,02613924
IER2	1785,3617	-1,1792106	0,02647155
CEMIP2	2377,80752	1,17279494	0,02666804
ELL2	1601,24604	1,22735224	0,02848527
DUSP4	222,298797	2,18753877	0,02906114
OSBPL3	860,048657	-1,3673434	0,02994598
UTP15	173,095807	2,29066008	0,03197661
CCL5	2026,12134	1,24301575	0,0325518
FAAH2	298,449296	-2,5103599	0,0333323
SH3GL2	103,805127	-4,2631558	0,03353995
INPP4B	2113,26632	-1,1142459	0,03373765
EHF	161,281302	-3,3877829	0,0370701
CCND3	1882,08622	-1,1856005	0,03778812
B4GALNT1	483,146422	2,16519917	0,03861167
PYM1	304,539556	-1,9266535	0,03861167
ACAD9	383,775472	1,76924402	0,039614
ABCA7	213,718253	-2,5684765	0,039614
PDZK1	918,448624	-1,5712673	0,0399099
EHD1	604,20394	-1,3444919	0,0399099
ELF1	3104,75508	1,02810205	0,0399099
SDF4	414,30788	-1,6523531	0,04184225
CLEC11A	637,558046	-1,2631475	0,04184225
FAM177A1	2850,98605	1,21837764	0,04240422
LGALS1	346,820624	1,71263253	0,04240422
GPR35	1558,16415	1,29905856	0,044004
CRK	218,127513	2,0289101	0,044004
FLNA	1434,05505	-1,0033767	0,04626419
PPA1	1605,16899	1,21856702	0,04841171
FBLN5	442,201331	-1,6973372	0,04841171
GLIPR2	1179,23833	-1,0068281	0,04942632
PRMT5	712,26881	-1,2548093	0,04942632
DYNLT3	1254,96567	1,01998142	0,04948575
TEX10	227,305328	2,02356589	0,04989065

SKIN VS INTESTINE

**Genes**	**baseMean**	**log2FoldChange**	**padj**
CA6	520,266982	4,03669495	6,4533E-13
BAMBI	1080,83224	-2,9206153	1,2944E-12
RGS2	14586,1648	-2,3287062	4,0061E-11
CXCR4	27979,9487	-1,9763206	1,0557E-08
ENG	357,250473	-4,0541159	1,4654E-08
CYP26A1	1340,13483	-2,7721833	2,4643E-08
RGS1	12313,8901	-1,5419881	1,7579E-07
IL2RA	1356,35765	2,40505672	1,9219E-07
SMOX	355,157993	4,13543486	6,1546E-07
BCL6	2891,0085	-1,5398626	1,0108E-06
ARRDC3	2696,17725	-1,9700499	1,0198E-06
SPRY1	1952,82525	-2,1710979	1,0198E-06
ICAM1	3703,57879	1,74963093	1,162E-06
FOS	40286,9382	-2,2000406	1,7655E-06
GSTP1	989,457287	-2,3376011	3,0066E-06
DHRS3	5051,06257	-1,7480507	3,7904E-06

ING4	442,697643	2,54622845	8,4849E-06
ATF3	1578,95046	-3,189011	1,1912E-05
ADPRS	633,986092	2,2383083	2,0326E-05
CD2	934,563313	-2,2868541	2,8649E-05
ENPP2	176,13227	4,15223368	3,112E-05
GNG13	214,345321	-3,1947931	4,1534E-05
LRRC25	108,401134	-5,830171	4,1534E-05
SDC4	4280,60707	1,62611303	5,0619E-05
PLK2	639,559035	-2,3326652	5,4607E-05
CD38	576,214292	-2,8131655	7,3852E-05
ILF3-DT	248,879305	-3,0559398	0,00013302
GBP5	2957,86469	-1,3834957	0,00013822
ABHD15	875,484189	-1,8570844	0,00016165
LAIR1	460,329422	-2,0892455	0,00030172
STX11	592,778357	3,66516743	0,00034851
ALDOC	14927,4636	-1,2338337	0,000349
SUSD3	1136,27496	-2,0106102	0,00038747
LGALS1	346,820624	2,41230474	0,00062525
SLC1A5	226,71986	2,98643901	0,00088403
RARG	658,401845	2,26033018	0,00136367
FHL3	428,537943	-2,0979005	0,00163363
CLU	154,874198	4,93511869	0,00163363
KLF10	686,628931	2,6235662	0,00163363
CD69	9554,77394	-1,3189733	0,00163363
NFKBID	1568,49958	1,37591036	0,00163363
ID2	30019,6166	-1,3106212	0,00165769
TNFSF13B	1130,67605	-1,4330422	0,00189709
S1PR1	5549,98838	1,63406008	0,00227934
CFH	2524,67503	1,24940931	0,00240297
NKIRAS1	913,634414	-1,6141841	0,00240297
TNFAIP3	9711,99037	-1,3189251	0,00253684
DUSP1	38824,3054	-1,5726598	0,00268957
ELOA	1467,65767	1,54734784	0,00292962
SNX30	232,028899	-3,4677275	0,00313785
TNFSF14	1355,40127	1,65917664	0,00328934
SDF4	414,30788	2,10618725	0,00335914
EGR2	327,472152	2,25743418	0,00351665
CDCA4	298,531132	2,722042	0,00384687
TMBIM1	1146,32262	1,52815101	0,00411396
TP53INP1	677,352772	-1,6390826	0,00432776
NGF	238,355429	-3,7972506	0,00457803
RORC	3605,17464	-1,2437204	0,00470494
NCR2	343,573911	-2,5413484	0,00485585
MFSD5	326,535735	2,77173762	0,00485585
HELB	322,243196	-2,4880595	0,00539142
PLAT	114,622371	-3,5113877	0,00547102
JUNB	2217,63237	-1,6310642	0,00549032
SVIL-AS1	331,539869	-1,9411327	0,00571073
LIF	823,166402	-1,5444787	0,00571073
LMNA	2087,46754	1,52459485	0,00599753
ABCG1	255,382284	2,16840304	0,00624766
CEP70	161,33386	-3,2890923	0,00651475
PRUNE1	590,191602	-2,2136624	0,00672306
EMP2	96,8373218	-5,4541578	0,00685621
BATF	2035,19591	1,57237505	0,00786774
KATNB1	448,922324	1,91656695	0,00798771
F13A1	182,466634	-4,6225384	0,00876372
WHRN	211,146794	-2,4467802	0,00894092
FAS	765,333181	2,19690194	0,00894092
CBFA2T2	753,357802	-1,519949	0,00908193
AFAP1L1	454,210698	1,73557446	0,00920436
E2F1	156,056049	3,7650787	0,00932419
CTSW	7408,21409	1,13538005	0,00941241
TGM2	1412,21676	-1,3127996	0,00977535

SLAMF1	336,260043	2,45312182	0,01036283
SPATA5	193,589919	-2,4929263	0,01036283
LDB2	467,427601	-1,7368242	0,01095121
RBBP8	369,545518	1,99950061	0,01127072
SOX4	959,670566	-1,3179463	0,01130279
EDC3	233,921502	-2,401907	0,01164782
ABCB1	597,609452	-1,9913616	0,01265782
ILVBL	330,032328	1,90300541	0,01352818
NINJ2	504,153665	-1,8282797	0,01359057
TGFBR1	329,753637	2,09948908	0,01483086
HDDC2	431,523207	-1,671994	0,01484697
KLF2	356,997878	-1,7782815	0,01484697
B3GNT7	4248,23076	-1,145376	0,0164115
ACAT2	2170,20474	1,17860226	0,0164115
DST	504,418975	-1,5793157	0,0164115
IFRD2	1248,70295	1,31460441	0,01732866
IMPDH1	211,416262	3,12174417	0,01809867
CCDC82	239,027388	2,07849621	0,01920427
CDC45	658,848087	1,74435462	0,01932615
NUP62	1217,96264	1,21710511	0,01947324
CEACAM1	507,516072	-1,6348783	0,02075767
ANKRD37	432,600986	-1,7214445	0,02087245
NFKB2	369,725042	1,75823738	0,02243041
DNAJC9	591,278689	1,37356031	0,02257253
NFKBIE	179,850844	2,5144402	0,02292031
PPP1R16B	287,059586	1,88085907	0,02303813
TMEM11	896,696186	1,4738287	0,023373
PDGFA	588,969999	1,84712086	0,0240457
COQ9	697,66646	-1,4391646	0,02421955
ZNF622	825,533585	1,38196125	0,02533137
ZFP36	14561,4746	-1,3434289	0,02568945
PRKAB1	1268,63716	-1,1082824	0,02581651
CREB3L2	591,814961	-2,0063243	0,02711795
SOCS2	544,510627	1,40974347	0,02711795
SEC31B	369,264095	1,75683516	0,0278905
CTDSP2	387,278137	-1,781162	0,02861291
PELO	385,176125	1,57333099	0,02958583
EXOC3	269,826081	-2,1821587	0,02958583
PEG10	796,240987	-1,4669994	0,02958583
EFHC1	261,802378	-2,0726574	0,03016157
PER2	357,729981	1,87362461	0,0306833
LDLRAD4	629,400404	-1,6239348	0,0309273
FEN1	921,511446	1,2888583	0,03250414
ANKRD49	491,94621	1,70339646	0,03250414
RAD51	378,471189	1,78799537	0,03360988
CYBRD1	276,981721	1,99604056	0,03363015
RGS14	229,770082	-2,0359367	0,03363015
CDIP1	340,444368	-1,6930277	0,03428613
PIGQ	305,043318	2,11496309	0,03435917
BAZ1A	350,744007	1,65775571	0,03528193
RPAP1	284,41509	-2,3590501	0,03653683
EIF3B	503,381251	1,37357083	0,03769169
TNFRSF18	914,778228	1,76069649	0,03844646
F2R	288,26695	-2,0003793	0,0395084
PIAS3	250,84387	1,89301496	0,03957143
H2BC21	1089,07759	-1,0815207	0,03957143
PDP1	227,620392	2,04409198	0,03957143
DUSP6	445,864136	-1,5788359	0,03957143
PITRM1	972,242766	-1,2979164	0,03972737
KRT81	654,027839	-1,4174393	0,04061915
CNPPD1	428,218152	1,6481037	0,0406621
HAT1	1190,2206	1,07461468	0,04071707
CXCL2	1671,58407	-1,9143917	0,04147561
SESN1	3693,06395	-1,1146243	0,04165242

TBL3	496,644222	1,5095564	0,04165242
P3H1	784,52554	-1,2721463	0,0445829
HMG20A	884,298353	-1,212394	0,0445829
B3GALNT1	185,288739	2,00040349	0,04532992
RNF122	379,318062	-1,7642711	0,04532992
GMNN	736,323875	1,3498952	0,04831887
PDE4B	1236,56876	-1,2308729	0,04932739

SKIN VS LIVER

Genes	baseMean	log2FoldChange	padj
IL2RA	1356,35765	3,24730592	8,6017E-14
SRPX	203,298741	5,97462339	6,5963E-12
RGS2	14586,1648	-2,2663945	1,9868E-10
EGR2	327,472152	3,99488353	3,9773E-10
PEG10	796,240987	-3,0242586	1,0277E-09
ENPP2	176,13227	5,78557777	5,7904E-09
ILF3-DT	248,879305	-3,9588698	5,7159E-08
LGALS1	346,820624	3,27925226	2,2832E-07
SMOX	355,157993	3,99662115	1,0677E-06
BCL6	2891,0085	-1,5116291	1,9595E-06
SAMSN1	1356,93756	2,71733429	1,8298E-05
ENG	357,250473	-3,3053009	1,9523E-05
LMNA	2087,46754	2,02704132	4,1818E-05
LZTFL1	1554,22445	-1,8442017	4,1818E-05
BAMBI	1080,83224	-1,9230865	4,1818E-05
DUSP1	38824,3054	-1,9372835	4,7605E-05
ICAM1	3703,57879	1,53350598	5,572E-05
FHL3	428,537943	-2,4707962	6,0221E-05
IRF7	2180,41811	1,86598214	7,0255E-05
TRAF4	523,126249	2,36664769	7,0255E-05
H2BC21	1089,07759	-1,6566429	0,00012002
CDKN1A	922,559262	2,21032467	0,00025854
AGO2	510,293183	2,08495243	0,00025854
NFKBID	1568,49958	1,5142737	0,00026303
RORC	3605,17464	-1,4750016	0,00027987
ARRDC3	2696,17725	-1,5915588	0,00027987
DNMBP	147,376865	4,810403	0,00027987
CYP26A1	1340,13483	2,08479776	0,00029054
SRSF7	9309,1686	1,11918129	0,00033302
CA6	520,266982	2,22643889	0,00054453
MT-RNR1	134422,71	-1,0356713	0,00067202
ZIK1	203,949706	3,27545043	0,00077899
TTN	3698,18809	-2,0503186	0,00082987
KLF2	356,997878	-2,1946623	0,00085828
RGS14	229,770082	-2,7245419	0,00096446
BCOR	791,493521	1,74956512	0,00102419
DHCR7	1514,12027	1,35969664	0,00104773
CXCR4	27979,9487	-1,3012827	0,00110701
TXNIP	4033,70901	-1,8100544	0,00121268
CHAD	435,542027	-2,4631652	0,00148115
SDC4	4280,60707	1,36832174	0,00160231
HPCAL1	872,974173	1,7087688	0,00180667
SUSD3	1136,27496	-1,8378337	0,00180667
RANBP1	1580,57421	1,28750154	0,00180667
SLAMF1	336,260043	2,79406987	0,00185047
SNX30	232,028899	-3,5485783	0,00185047
COQ9	697,66646	-1,7718255	0,00185047
PCDH9	1811,62607	-1,3008433	0,00205152
LRRC25	108,401134	-4,7650991	0,0021653
NLRP3	309,288225	2,64801967	0,00225545
SHISA2	1915,73981	1,912769	0,00225545
FAM3A	225,984836	-2,6989981	0,00235112
INPP4B	2113,26632	-1,4026121	0,00235516
HMG20A	884,298353	-1,5882274	0,00235516

CXCL2	1671,58407	-2,4733345	0,00248861
PDGFA	588,969999	2,21743861	0,00248861
AGT	1952,9033	-1,5753904	0,00271208
VWA5A	923,200613	-2,3244936	0,0029202
SLC43A1	289,217978	2,34985359	0,00326893
JUNB	2217,63237	-1,6695379	0,00367205
SPRY1	1952,82525	-1,4889777	0,00384845
GFRA2	1489,73405	-1,6304211	0,00384845
TOB1	20403,0785	-1,18459	0,00384845
TBXAS1	262,88434	2,39306336	0,00474314
ELOA	1467,65767	1,47133773	0,00477967
C19orf48	2192,61259	1,62852448	0,00551969
WDR3	338,468511	-2,0269795	0,00574658
ZNF430	350,222448	2,4020209	0,00583904
COG1	350,322106	-2,2070878	0,00588744
NCAPG2	1968,112	-1,2120778	0,00593027
ABCA7	213,718253	-3,1025356	0,00593027
IL7R	15019,2636	-1,0186823	0,00613718
ADAM19	1186,8218	1,50278854	0,00643295
MFSD5	326,535735	2,68242912	0,00643295
DUSP14	252,031515	2,94736476	0,00643295
SEC23B	2240,35147	1,15698461	0,00643295
PAF1	1106,25671	1,53794644	0,00643295
BRD2	1587,59153	1,40527193	0,00648333
FAM120AOS	369,044639	2,34905048	0,00689901
NEIL2	270,41372	-2,5204671	0,0069724
CCL20	407,146512	-2,5337004	0,00762798
HAT1	1190,2206	1,27019366	0,00762798
LETMD1	408,707995	-1,9054789	0,0077316
S100A13	424,932696	-1,9650031	0,00776574
GRPEL1	1976,06119	1,1261912	0,00789194
SMC1A	1286,63757	1,26954938	0,00794176
SRRT	673,785712	1,51990769	0,00801094
LIMS1	491,957613	-1,9135203	0,00814168
POR	2660,90893	1,13572494	0,00823553
GIMAP4	251,083514	-2,3551185	0,00827854
IL17RE	229,211692	-2,440134	0,00836721
FOS	40286,9382	-1,4288883	0,00854526
BCAS3	616,07274	-2,0132064	0,00854526
DST	504,418975	-1,6454223	0,00864179
HROB	237,372983	3,18755695	0,00899902
EOLA1	634,619704	-2,0322101	0,0090544
METTL3	862,205949	-1,3484167	0,00991993
RALGAPA2	207,453944	-2,4901068	0,00995643
CDC45	658,848087	1,81901917	0,01021548
GATA3	612,453122	1,61688389	0,01080502
PER2	357,729981	2,0486157	0,01085799
DDX3Y	2207,88003	1,00542701	0,01087031
DENND2D	920,875827	-1,6372681	0,01148983
TAGAP	1644,33914	-1,3253132	0,01148983
MGME1	417,450003	-1,7126875	0,01148983
BCAS2	2889,93248	-1,122345	0,01153652
KHDRBS2	268,847796	-2,0956877	0,01153652
S100A6	5016,07716	1,07447416	0,01153673
LDB2	467,427601	-1,6934502	0,01153673
ANKRD13A	279,388235	2,0240555	0,01153673
ACAT2	2170,20474	1,18103323	0,01327903
CREB3L2	591,814961	-2,1097611	0,01348484
CTSW	7408,21409	1,07984911	0,01348484
NGF	238,355429	-3,3768482	0,0141244
MAP2K3	633,684773	1,50117627	0,01441093
KIAA0087	168,604602	-2,4407861	0,01460757
PTPN6	2165,3576	-1,5772324	0,01467801
PELO	385,176125	1,65557382	0,01514778

RFWD3	370,290403	1,70625609	0,01557751
GGH	383,273892	2,06981873	0,01695226
EIF5	4624,95716	1,19730753	0,01710199
TMEM101	558,301246	-1,6617582	0,01722284
CRK	218,127513	2,21950653	0,01722284
ADA	766,636378	1,53896041	0,01894111
RBM6	2104,33735	-1,2972198	0,01908459
TMEM168	233,281005	-2,2666393	0,01927555
ATF3	1578,95046	-2,0369115	0,01930067
SESN1	3693,06395	-1,2023013	0,01930067
ABCA1	454,49357	-1,6870643	0,01930067
FLAD1	820,184447	-1,5265506	0,02016806
PUM1	1421,58819	-1,3327139	0,02139062
NCOA7	2785,42698	1,04682359	0,02170629
INPP1	403,994254	1,69940179	0,02209614
PPP1R9A	1657,51028	-1,1641026	0,02209614
PLPP1	1044,83146	1,2401962	0,02214861
FAS	765,333181	1,96884816	0,02237422
ZYX	256,996084	2,5391008	0,02317745
PECAM1	3521,81345	-1,0369026	0,02317745
CBFA2T2	753,357802	-1,3601928	0,02318779
TSPYL2	2202,56265	1,21958807	0,02355266
SFXN3	321,623092	2,51018214	0,02442979
CCND3	1882,08622	-1,2202547	0,0245287
S1PR1	5549,98838	1,29403493	0,02466107
RAE1	1227,31064	1,27540729	0,02492148
PGAM1	1204,23168	1,0972928	0,02495859
RNF168	518,11745	1,61445945	0,02518761
DUSP2	4036,80544	-1,5255725	0,02576054
RBM18	490,898481	-1,5068335	0,02595655
GRWD1	692,610417	1,71752478	0,02605154
STARD4	521,285234	2,14304578	0,02753004
PSMB5	1277,8424	1,18329527	0,02989892
HMGA1	1331,08137	1,27360278	0,03016024
MTMR1	192,357865	-2,5291456	0,03016024
CHST9	421,482333	-1,797723	0,03150336
MAT2A	457,830419	1,60267638	0,03150394
XPO5	228,986722	1,920725	0,03192988
RORA	1392,14296	1,17932932	0,03192988
WWP2	769,633946	-1,2889126	0,03248483
ERCC2	320,779736	-1,9620657	0,03310154
ANKRD46	164,403118	-2,2804653	0,03334887
VIM	616,06606	1,47905663	0,03435161
PARN	967,454736	-1,1751014	0,03435161
P4HA1	1237,68259	1,19657306	0,03442609
XCL1	3765,90302	1,43268946	0,03532598
TNFRSF4	302,039051	1,85479259	0,03532598
THUMPD3-AS1	1015,69522	1,39122477	0,03536924
MARF1	197,14768	-2,270842	0,03539351
ALOX5AP	4612,00829	1,04848107	0,03552234
PHF5A	471,817163	1,50400132	0,03552234
LAIR1	460,329422	-1,4147448	0,03552234
CRYBG1	669,23064	1,68774984	0,03563619
SLC38A2	1042,34933	1,11001298	0,03563619
MHENCR	368,925877	-1,6582022	0,03620882
EIF4H	1275,15362	1,10131441	0,03660177
RNF122	379,318062	-1,772555	0,03710834
ADPRS	633,986092	1,33104055	0,03836314
MAP7D3	263,778857	-1,8260039	0,03891093
CYHR1	231,628172	-1,9939011	0,03891093
ATP13A2	119,404169	-3,6330861	0,03995134
CRY2	869,17872	1,36357013	0,03995134
TMEM38A	175,741178	-2,912113	0,04013032
CSK	397,85603	-1,7109529	0,04117329

DIP2B	293,770083	-1,9952008	0,04197375
SNX27	614,605161	-1,3443136	0,04261126
CAMK2G	452,597695	-1,6299085	0,04262451
H2BC8	375,166267	-2,671999	0,04417133
RBM41	204,379703	-2,2782917	0,04560686
GNG2	1529,77112	1,11697838	0,04596068
SERTAD1	270,659806	2,3612916	0,0464408
PARVB	153,009281	2,39536657	0,04672534
ZFP36	14561,4746	-1,2136372	0,04692107
ARHGEF39	279,085254	-1,9654903	0,04740708
PDE9A	231,687292	2,41768576	0,0477178
CHFR	504,48839	-1,4639242	0,04789995
DENND4A	586,196965	1,39233939	0,04941576

SKIN VS LUNG

**Genes**	**baseMean**	**log2FoldChange**	**padj**
RGS2	14586,1648	-2,4704863	1,3495E-12
CYP26A1	1340,13483	-3,385217	1,3495E-12
ENG	357,250473	-4,4640619	1,7427E-10
RGS1	12313,8901	-1,6586875	1,2825E-08
DHRS3	5051,06257	-2,0539008	1,7677E-08
PLK2	639,559035	-2,9408742	5,349E-08
BAMBI	1080,83224	-2,3116591	1,4089E-07
CXCR4	27979,9487	-1,8418628	1,6232E-07
SLC2A3	14612,0042	-1,5911254	8,4121E-06
ICAM1	3703,57879	1,6701541	8,5246E-06
MFSD5	326,535735	3,85705867	1,2115E-05
NKIRAS1	913,634414	-2,079171	1,6248E-05
EMP3	3326,75679	1,69515517	2,6517E-05
BCL6	2891,0085	-1,3840795	3,1445E-05
SPRY1	1952,82525	-1,9394152	3,882E-05
IL32	6197,12845	1,35244931	6,8918E-05
TMBIM1	1146,32262	1,93373107	7,1698E-05
AMPD2	758,21677	-1,7757514	0,00042858
ELOA	1467,65767	1,76854127	0,00042913
SDC4	4280,60707	1,47185272	0,0007795
SDF4	414,30788	2,32658946	0,00112456
EGR2	327,472152	2,50145065	0,00112456
CD2	934,563313	-1,9635827	0,00124263
LAIR1	460,329422	-1,977846	0,00126715
SLC25A20	294,01542	2,2782089	0,00196942
ID2	30019,6166	-1,3218043	0,00202108
WDPCP	243,040003	2,47005043	0,00264202
PAF1	1106,25671	1,69436136	0,00286577
CBFA2T2	753,357802	-1,7047391	0,00301493
FOS	40286,9382	-1,6109058	0,00312942
BIRC3	4067,00636	1,17766643	0,00339716
SERPINF1	256,639875	2,73395976	0,00339716
ADPRS	633,986092	1,75363961	0,00351866
ARRDC3	2696,17725	-1,3944937	0,00363091
NGF	238,355429	-3,8884165	0,00436167
EOLA1	634,619704	-2,2273327	0,00490885
ZNF331	6695,53309	-1,2321284	0,0055953
ILF3-DT	248,879305	-2,4784108	0,00745636
ALOX5AP	4612,00829	-1,2872858	0,00847316
KATNB1	448,922324	1,96870625	0,00847316
ABCG1	255,382284	2,1852027	0,00858783
PRMT9	3083,18465	-1,4670473	0,00895807
PYHIN1	1222,14506	-1,3935555	0,00933576
ASB2	1215,27413	-1,311951	0,00979061
TP53INP1	677,352772	-1,5774236	0,00994602
ATF3	1578,95046	-2,2869788	0,01001341
CTSW	7408,21409	1,16479768	0,01055613
C3orf52	391,243546	-2,229256	0,01067858

PLAT	114,622371	-3,4209965	0,01067971
BIN1	662,796883	1,72944478	0,01208517
CCNH	2239,38879	-1,2029454	0,01208517
LMNA	2087,46754	1,48013983	0,01216921
ANXA1	3459,6797	1,59333416	0,01216921
CDC42SE1	3620,67825	1,0220493	0,01348545
PLIN2	12927,8937	-1,0963349	0,01410121
PLAAT4	1535,89569	-1,1139257	0,01500943
BATF	2035,19591	1,52561576	0,01600453
F13A1	182,466634	-4,51836	0,01658794
ABCB8	268,616873	2,79327243	0,01680859
SC5D	1814,9737	-1,6067921	0,01680859
KRT81	654,027839	-1,6265718	0,01680859
SPATA5	193,589919	-2,4565784	0,01744254
RARG	658,401845	1,88735948	0,01767587
PPP1R15A	5435,67561	1,14688211	0,01786858
TNF	1223,14578	1,70736361	0,01841668
LDB2	467,427601	-1,7002971	0,01866691
TNFSF13B	1130,67605	-1,2175904	0,0188648
CPSF1	395,681234	1,96350138	0,01948744
SLC43A1	289,217978	2,08604042	0,02014611
KLHL24	1492,89845	-1,0682803	0,02450999
TGFBI	180,311589	-2,5644935	0,02455831
TRPV2	685,98117	1,47982903	0,02561783
NCR2	343,573911	-2,2484401	0,0263798
CPXM1	312,707507	1,91426514	0,02702776
LPIN1	888,281269	-1,3630548	0,02770584
ANKRD37	432,600986	-1,7191472	0,02868711
ITM2C	5617,76862	1,18229242	0,02951297
CNPPD1	428,218152	1,7814853	0,03046652
TNFSF14	1355,40127	1,38916422	0,03169466
E2F1	156,056049	3,42018624	0,03286711
EFCAB8	274,423098	-2,9335249	0,03305675
ABHD15	875,484189	-1,314659	0,03394225
PDGFA	588,969999	1,82654645	0,03673386
AGT	1952,9033	-1,3036319	0,03710649
KLF10	686,628931	2,05220481	0,03866023
EHD4	1028,76882	1,47606168	0,04027214
SNX30	232,028899	-2,816203	0,04173628
ZNF488	417,074949	2,02389655	0,04173628
SAP130	284,547176	2,11723964	0,0449124
DEDD2	479,613427	1,55460906	0,04548641
CD38	576,214292	-1,8627994	0,04560204
DHX38	452,598843	1,88739979	0,04561588
IL27RA	1055,71884	1,40857419	0,04561588
PRMT6	144,410325	2,6162462	0,04756281
ERCC2	320,779736	-1,9998792	0,04822603
